# Multidimensional encoding of brain connectomes

**DOI:** 10.1038/s41598-017-09250-w

**Published:** 2017-09-13

**Authors:** Cesar F. Caiafa, Franco Pestilli

**Affiliations:** 1 Department of Psychological and, Brain Sciences Indiana University Bloomington, IN, 47405 USA; 2 Department of Intelligent Systems, Engineering Indiana University Bloomington, IN, 47405 USA; 3 Department of Computer Science, Indiana University Bloomington, IN, 47405 USA; 4 Program in Neuroscience Indiana University Bloomington, IN, 47405 USA; 5 Program in Cognitive Science Indiana University Bloomington, IN, 47405 USA; 6 School of Optometry Indiana University Bloomington, IN, 47405 USA; 7 Indiana Network Science Institute Indiana University Bloomington, IN, 47405 USA; 8 Instituto Argentino de Radioastronomía (IAR), CONICET CCT, La Plata Villa Elisa, 1894 Argentina; 9 Facultad de Ingeniería - Departamento de Computación, UBA Buenos Aires, C1063ACV Argentina

## Abstract

The ability to map brain networks in living individuals is fundamental in efforts to chart the relation between human behavior, health and disease. Advances in network neuroscience may benefit from developing new frameworks for mapping brain connectomes. We present a framework to encode structural brain connectomes and diffusion-weighted magnetic resonance (dMRI) data using multidimensional arrays. The framework integrates the relation between connectome nodes, edges, white matter fascicles and diffusion data. We demonstrate the utility of the framework for *in vivo* white matter mapping and anatomical computing by evaluating 1,490 connectomes, thirteen tractography methods, and three data sets. The framework dramatically reduces storage requirements for connectome evaluation methods, with up to 40x compression factors. Evaluation of multiple, diverse datasets demonstrates the importance of spatial resolution in dMRI. We measured large increases in connectome resolution as function of data spatial resolution (up to 52%). Moreover, we demonstrate that the framework allows performing anatomical manipulations on white matter tracts for statistical inference and to study the white matter geometrical organization. Finally, we provide open-source software implementing the method and data to reproduce the results.

## Introduction

A fundamental goal of neuroscience is to develop methods to understand how brain networks support function and behavior in individuals across human populations^1–4^. The recent increase in availability of neuroimaging data and large scale projects has the potential to empower new ways of discovery by studying large populations of human brains^5–23^. Exploiting these large-scale data sets will require convergent efforts in advancing measurement methods, data representation frameworks, as well as computational algorithms and theory^24,25^.

Recent advances in measurement methods and computational algorithms are shifting the study of the white matter and brain networks beyond qualitative characterization (such as camera lucida drawings), toward structural and functional quantification^26–31^. Tractography and diffusion-weighted magnetic resonance imaging (dMRI) are the primary methods for mapping structural brain networks and white matter tissue properties in living human brains. Using these methods we have learned much about the macrostructural organization of the human brain, such that network neuroscience has become one of the fastest-growing scientific fields^3,27,28,30,32–40^.

**Figure 1:**
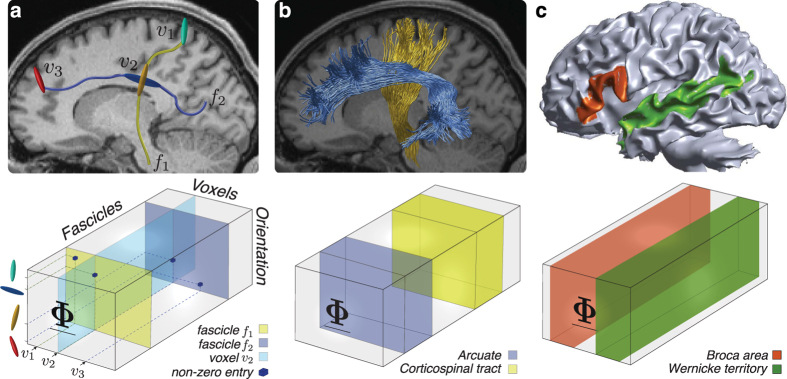
Connectome encoding using multidimensional arrays. (**a**) **Top**. Two white matter fascicles (*f*_1_ and *f*_2_) and three voxels (*v*_1_, *v*_2_ and *v*_3_). **Bottom**. Tensor encoding of fascicles’ spatial and geometrical properties. Yellow *f*_1_, dark blue *f*_2_, cyan *v*_2_. Non-zero entries in $\underline{{\boldsymbol{\Phi }}}$ indicate fascicles orientation (1^st^ mode), position (voxel, 2^nd^ mode) and identity (3^rd^ mode). (**b**) **Top**. Two major human white matter tracts (connectome edges). The corticospinal tract and Arcuate fasciculus. **Bottom**. Tensor encoding of connectome edges. The corticospinal tract and Arcuate fasciculus are encoded as collections of frontal slices–blue and yellow subtensors. (**c**) **Top**. Two human cortical areas (connectome nodes). Wernicke’s territory and Broca’s area. **Bottom**. Tensor encoding of connectome nodes. We show examples of a large temporal area comprising also Wernicke’s territory and Broca’s area encoded as collections of lateral slices–red and green subtensors (areas defined using Freesurfer^43–45,130^).

Tractography algorithms use dMRI data to estimate the three-dimensional trajectory of neuronal axons bundles wrapped by myelin sheaths–the white matter fascicles. Fascicles are normally represented as sets of brain coordinates, with coordinates segments spanning anything between 0.01 to 1 *mm* in length (Fig. 1a **top**). Fascicles have historically been clustered into anatomically cohesive groups called white matter tracts. The largest of these tracts have associated names–such as the corticospinal tract (CST) and the arcuate fasciculus (Fig. 1b **top** ^41,42^). White matter tracts communicate between cytoarchitectonically and functionally distinct areas–such as Broca’s or Wernicke’s areas involved in human language processing (Fig. 1c **top** ^43–45^). White matter tracts and brain areas together compose a large-scale network called the connectome^46^. Within this network, white-matter tracts represent communication pathways (the edges; Fig. 1b **top**) and brain areas units of information processing (the nodes; Fig. 1c **top**).

We propose a connectome encoding framework that integrates models of white matter fascicles anatomy, microstructural tissue properties as well as the dMRI measurements. The framework encodes altogether connectome edges, nodes as well as the associated dMRI data using multidimensional arrays–also called tensors^47–50^. Below, we introduce the framework and show four applications. First, we use the framework to implement efficiently methods for connectome evaluation. Second, we use the framework to perform a large scale tractography evaluation (13 tracking algorithms, 1,490 brain connectomes, three different data sources^51–55^). Finally, we present two additional applications by describing how the framework can be used to perform efficiently statistical inferences on brain connections and white matter tracts using the recently introduced virtual lesion method^52,56^ and to chart the reliability and reproducibility in the estimates of the geometrical organization of the human white matter^57–59^. We provide open source software^60,61^ implementing the encoding framework at http://www.github.com/brain-life/encode and data to reproduce the analyses at http://hdl.handle.net/2022/21480.

## Results

We present a method to encode the anatomical properties of connectome edges and nodes into multidimensional arrays^47^ (see Supplementary Methods, section 1.1). The encoding scheme maps fascicles into the three dimensions of a sparse array $\underline{{\boldsymbol{\Phi }}}$ (Fig. 1a **bottom**). The first dimension of $\underline{{\boldsymbol{\Phi }}}$ (1^st^ mode) encodes fascicles orientation along their trajectory. Where single nodes in a fascicle are encoded as non-zero entries of the sparse array (see dark-blue cubes in Fig. 1a **bottom**), and full fascicles as complete frontal slices (yellow and blue in Fig. 1a **bottom**). The second dimension (2^nd^ mode) encodes spatial position within the brain, that is voxels. Slices in this dimension represent single voxels (see cyan slice in Fig. 1a **bottom**). The third dimension (3^rd^ mode) encodes fascicles, or better the indices of each fascicle within the connectome. We show that connectome edges (a white matter tract) is an ensemble of fascicles that can be represented by a set of frontal slices in (Fig. 1b **bottom**). Once these slices are reorganized (permuted) they come together to represent a white matter tract. Blue and yellow frontal volumes (technically called sub-tensors) in Fig. 1b **bottom** correspond to the encoded representation of the Arcuate Fasciculus and Corticospinal Tract, reproduced in Fig. 1b **top** in their natural brain space. Also, connectome nodes (an ensemble of voxels) are encoded in $\underline{{\boldsymbol{\Phi }}}$. For example, Fig. 1c **bottom** shows the lateral sub-volumes encoding the voxels for Broca’s area (red) and Wernicke’s territory (green; regions also reproduced in their natural brain space in Fig. 1c **top**.)

Multidimensional encoding of connectomes provides a variety of computational opportunities. This is because direct array operations can be applied globally to connectomes. For example, fascicle search, area to area mapping, charting brain connections or fascicles crossing angles become trivial operations, such as finding indices in the array $\underline{{\boldsymbol{\Phi }}}$. Below, we demonstrate four applications involving such operations. Section 2.3.1 of the Supplementary Material describes in more detail advantages and disadvantages of the encoding method.

### First application: Efficient connectome evaluation

It has been recognized that estimates of brain connectomes can differ substantially depending on the tracking method and data type^52,58,59,62^. Such differences motivated measuring accuracy for brain connectomes in individual brains in order to identify the best fitting connectome model before further studying its properties^52,62^.

A few methods to evaluate connectomes and compute errors have been proposed recently^52,63,64^. One of these methods, the Linear Fascicle Evaluation algorithm, or LiFE^52^, computes the error of a connectome in predicting the diffusion signal. LiFE takes as input the set of white-matter fascicles generated using tractography and returns as output the subset of fascicles that predict the dMRI measurements with smallest error (see^52^ and **Methods**). LiFE predicts diffusion measurements (vector **y**, Equation 4) in individual brains by combining the diffusion prediction from individual fascicles in a connectome (columns of matrix **M**, Equation 4) as described in Supplementary Fig. 2c. The LiFE model is fit to the data by assigning weights to the fascicles in the connectome (entries in vector **w**, Equation 4) via a non-negative least-squares method. We show that the LiFE model based on matrix **M** (hereafter referred to as LiFE_M_), can be accurately approximated using tensor decomposition and the framework introduced in Fig. 1 (see Supplementary Results). Hereafter, we refer to the LiFE model represented by multidimensional arrays as LiFE_T_.

**Figure 2:**
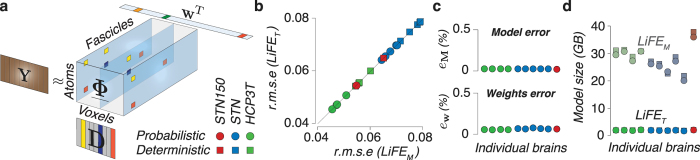
Tensor decomposition of the Linear Fascicle Evaluation method. (**a**) The tensor decomposition model, LiFE_T_ (see Supplementary Section 2.1 for details). LiFE_T_ uses a dictionary (matrix **D**) of precomputed diffusion predictions in combination with the sparse tensor, $\underline{{\boldsymbol{\Phi }}}$, and a vector of fascicles weights (**w**) to model the measured dMRI signal (matrix **Y**). (**b**) **Comparison of the error in predicting diffusion**. Scatter plot of the global r.m.s error (${\bar{e}}_{rms}$; equation (8), **Methods**) in predicting diffusion measurements for LiFE_M_^52^ and LiFE_T_ in ten brains, three dataset (HCP3T, STN and STN150) and two tracking methods (tensor-based deterministic and probabilistic tractography). The r.m.s is virtually identical. (**c**) **top** LiFE_T_ error in approximating the LiFE_M_ matrix (*e*_**M**_; Equation (9); **Methods**) computed for ten brains (HCP3T, STN and STN150 datasets, probabilistic tractography, *L*_*max*_ = 10). **Bottom**. Error (*e*_w_; Equation (10); **Methods**) of LiFE_T_ in recovering the fascicle contributions (vector **w**) assigned by LiFE_M_. (*N* = 10, probabilistic tractography *L*_*max*_ = 10) (**d**) **Model compression**. Measured size of LiFE_M_ (matrix **M**) and the decomposed model, LiFE_T_, (tensor $\underline{{\boldsymbol{\Phi }}}$ and matrix **D**; *N* = 20). Matrices and tensors all stored using double floating-point precision avoiding zero entries^70,71^.

Figure 2a depicts LiFE_T_, where the diffusion measurement (matrix **Y**, Equation (20) in Supplementary material) is factorized into: (1) a dictionary matrix **D** in which each atom (column) represents the precomputed diffusion prediction for a specific fascicle orientation, evaluated at all gradient directions (**θ**, see Equation (17) in Supplementary material), (2) the sparse array $\underline{{\boldsymbol{\Phi }}}$ (Fig. 1a **bottom**.) and (3) a vector of fascicle weights **w**. Supplementary Results, Section 2.1 provides additional details on the decomposition method.

We measured the accuracy of LiFE_T_ in approximating LiFE_M_ using three publicly available data sets: STN, STN150 and HCP3T^52–55,65^. To do so, we built connectomes in ten individual brains using both, probabilistic^66,67^ (CSD, *L*_*max*_ = 10 and deterministic^68,69^ tractography, see **Methods**). We report three main results showing that given a sufficient number of dictionary atoms (*L* > 360 in **D**; Supplementary Fig. 2d **)**: (1) the global r.m.s. error (Equation 8) in predicting diffusion is virtually identical between LiFE_M_ and LIFE_T_ (Fig. 2b). (2) LIFE_T_ approximates the LiFE_M_ matrix (**M**) accurately. Specifically, the Frobenius norm-based relative error, *e*_**M**_, is less than 0.1% (Fig. 2c **top**; **Methods**, Equation 9). (3) The fascicles weights assigned by LiFE_M_ and LIFE_T_ are virtually identical (Fig. 2c **bottom**, <0.1%). The relative error between weights estimated by LiFE_M_ and LiFE_T_, was computed using the *l*_2_-norm (**Methods**, Equation 10). We also show that by increasing decomposition resolution (*L*) the difference in r.m.s., as well as *e*_**M**_ and *e*_**w**_ decrease (Supplementary Fig. 2g,h and i).

Importantly, LiFE_T_ requires a fraction of the memory used by LiFE_M_. To show this, we measured the size of the computer memory used by matrix **M** in the LiFE_M_ model (**Methods**, Equation 4) and compared that to the total memory used by arrays $\underline{{\boldsymbol{\Phi }}}$ and **D** in the LiFE_T_ model (Equation (20) in Supplementary material). Figure 2d shows measurements in gigabytes for 20 connectomes (500,000 fascicles each, two tracking methods) in ten subjects from the three data sets. Whereas LiFE_M_ can require up to 40 GB per connectome, the decomposed model LiFE_T_ requires less than 1 GB, a 40x compression factor. All calculations were performed using double precision floating point and sparse data format^70,71^. See Supplementary Fig. 2 and Supplementary Results, Section 2.6 for details on the effect of the number of gradient directions (*N*_θ_) and connectome fascicles (*N*_*f*_) on memory consumption.

### Second application: Large-scale analysis of quality and reproducibility of tractography

The availability of multiple tracking methods and data types can be both an opportunity or a burden for investigators interested in using them as biomarkers for health and disease^7,13,14,16,23,72,73^. In an ideal world, a single tracking method or data type would supersede all others. ﻿In practice, a single algorithm or data type superior to all the rest has not been identified﻿. Yet multiple algorithms or data can help depending on study goals and available measurements infrastructure. For example, when measuring patient populations or in developmental or ageing studies it might be necessary to measure at lower resolution given time constraints. In principle, higher directional and spatial resolution should be preferred to lower resolution one. Yet, to date we do not have computations to relate data quality and resolution or tractography quality and flexibility to what it is possible to map of the human connectome.

We used LiFE_T_ to perform a large-scale evaluation of the reproducibility of connectome estimates in individual brains to identify the degree to which estimates depend on data quality and tractography. To do so, we generated a total of 1,490 connectomes using thirteen different combinations of tracking methods and parameters on data from twelve individual brains and three sources. Specifically, we used data from (a) HCP3T (4 subjects, 1.25 *mm* isotropic spatial resolution, 90 diffusion directions^22^, (b) HCP7T (5 subjects, 1.05 *mm* isotropic spatial resolution, 60 diffusion directions^55^ and (c) STN (4 subjects, 1.5 *mm* isotropic spatial resolution, 96 diffusion directions^52^.

To test the quality and reproducibility of connectome estimates we generated ten connectomes for each individual brain and tracking method. We used both, probabilistic and deterministic tracking, based on either constrained spherical deconvolution (CSD) or the tensor model^67,69^ and generated 500,000 candidate fascicles. We also varied tracking parameters by estimating fiber orientation distribution functions using a range of CSD parameter values (*L*_*max*_ = 2, 4, 6, 8, 10, 12). Each one of these 1,490 candidate connectomes was then processed using LiFE_T_. LiFE_T_ identified optimized connectomes, that is, the subset of fascicles with non-zero weight^52^ and computed connectomes error in predicting the diffusion signal (r.m.s., Equation 7). We used this large set of statistically validated, repeated-measures connectomes to test the reproducibility of connectome estimates in individual subjects, as function of tracking method and data type (spatial resolution, signal-to-noise ratio (SNR), and number of diffusion directions).

We assessed quality using multiple measures. Connectome quality can be assessed in several ways. For example, the error of the connectome in predicting the diffusion signal can be measured to establish connectome quality^52,63,64^. In addition, connectome resolution, the number of fascicles supported by the data can also inform about connectome quality. Finally, the accuracy of the connectome fascicles can be estimated qualitatively by comparing the anatomical variability of known major white matter tracts estimated from the connectomes using atlases^42^. We established the reproducibility of these three measures across repeated connectome estimates within individual brains and across tracking methods, parameters and data types.

**Figure 3:**
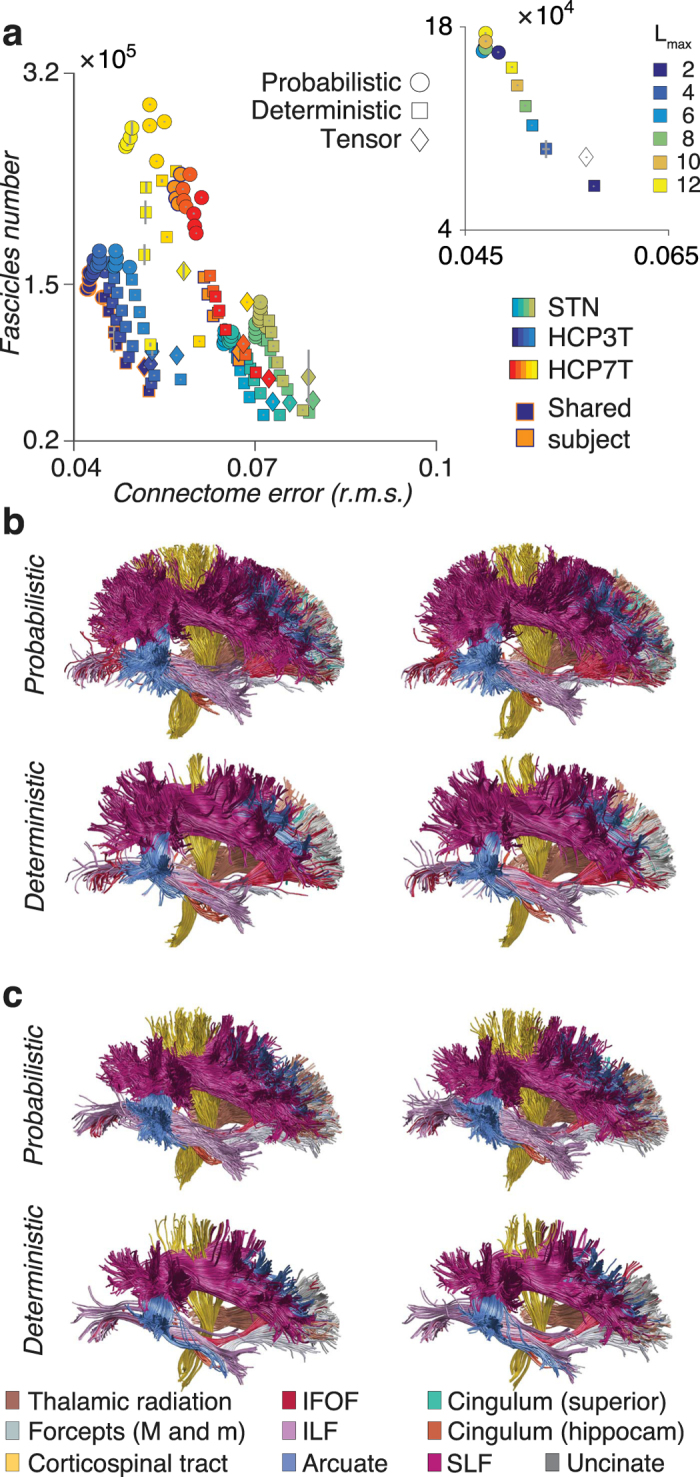
Connectome resolution and anatomical reliability as function of data and method. (**a**) Scatter plot of number of found fascicles and global r.m.s error in LiFE_T_ optimized connectomes (mean ±5 standard error of the mean, s.e.m., *N* = 1,490, *n* = 12 subjects, *m* = 10 repeated tracking, using either 13 or 9 different *L*_*max*_ values for either STN, HCP3T or HCP7T). Inset shows the relation between the number of found fascicles (ordinate) and r.m.s. error (abscissa) and *L*_*max*_ (color) in one subject from the HCP3T dataset. (**b)** Reproducibility of connectome anatomy. Twenty major human white matter tracts, two repeated estimates in a single subject probabilistic (top) and deterministic (bottom) tracking, HCP3T dataset. Tracts anatomy is very similar between repeated estimates when using a single tracking method (compare between columns, top and bottom). Estimated tracts anatomy differs within a single subject when the different tracking methods are used (compare between rows, left or right). (**c**) A different subject from the HCP3T dataset.

Figure 3a plots mean optimized connectome error and number of found fascicles (±5 standard error of the mean, s.e.m) for the three datasets: STN, HCP3T and HCP7T (1,490 connectomes). The plot shows a series of informative findings. First, data sets naturally cluster into groups, an effect mostly driven by the connectome error, the abscissa. Second, individual brains are nearly separable (along diagonals) both within and between datasets, such separation is largely independent of tracking method or parameters. Third, the number of found fascicles (connectome resolution) increases with the number of CSD parameters (*L*_*max*_), this is true in each data set, for both deterministic and probabilistic tracking but the effect is accentuated with deterministic methods (Fig. 3a inset). Fourth, connectome resolution and error are both extremely reliable. LiFE_T_ returns an almost identical number of found fascicles and connectome error across repeated tracking for a given set of parameter and tracking method (error bars are very small compared to the mean values). Fifth, probabilistic methods consistently show lower error in fitting the dMRI data and higher number of fascicles than deterministic models, this confirms previously reported results^52^.

Our results show that increasing dMRI data spatial resolution increases connectome resolution, despite differences in number of measured diffusion directions. To evaluate the impact of spatial resolution on the number of fascicles supported by the data, we first compared the number of fascicles assigned a non-zero weight by LiFE_T_ in a single subject between the 1.25 *mm* ^2^ and 1.05 *mm* ^3^ resolution (HCP3T and HCP7T respectively; blue and orange color in Fig. 3a). Results show a 46% (±5% s.e.m.) increase in number of fascicles with the higher resolution data set. An even larger increase in connectome resolution was measured across all subjects by comparing connectome resolution blocked by data resolution and averaged either across probabilistic (52% ± 3% s.e.m. across *L*_*max*_) or deterministic (50% ± 6% s.e.m. across *L*_*max*_) models. We computed this average by comparing only models common across data sets (i.e., *L*_*max*_ 2, 4, 6 and 8). Such 52% increase is well supported by the 68.7% increase in data volumetric resolution, and it is measured despite the decrease in number of measured diffusion directions in the higher resoution data (HCP7T: 60 directions, HCP3T: 90 directions). This demonstrates a profound impact of spatial resolution in mapping brain connectomes that goes beyond improvements due to directional resolution^55^.

We further performed a qualitative evaluation of the degree to which connectomes generated using different tracking methods and optimized with LiFE_T_ show reliable anatomical features. To do so, we segmented twenty major human white matter tracts using standard methods and atlases^42,74^. Figure 3b shows two examples of repeated tracts identified in one subject (HCP3T), using probabilistic (top) and deterministic (bottom) tracking. Results show high degree of anatomical similarity for tracts in LiFE_T_ optimized connectomes when using a single tracking method–compare left and right in the top or bottom panels. Conversely, results show anatomical differences within a single individual across tracking parameters–the LiFE_T_ optimization cannot change this result–compare top and bottom tracts. This reproduces previous results^52^. Figure 3c shows similar results for a different subject in the HCP3T data set. Importantly, by comparing two different subjects in Fig. 3b and Fig. 3c it is clearly possible to discriminate between brains based on the anatomical features of the connectomes. Supplementary Fig. 3b shows additional examples of major tracts anatomy estimated in individual subjects using repeated connectome measures. These plots allow to appreciate the degree of anatomical similarity within subjects given a single tracking method. Supplementary Fig. 3c shows multiple examples of major tracts anatomy estimated in individual subjects using different tracking methods and parameter sets. These plots also allow to appreciate the anatomical variability that different tracking methods introduced even within the same subject and data set by using different number of parameters for tracking.

### Third application: Statistical inference on white matter tracts

The concept of virtual lesion has been utilized in several contexts^56,75–78^. More recently, virtual lesions have been used to compute statistical evidence for white matter tracts by measuring the impact of removing entire sets of fascicles from individual whole-brain connectomes^52^.

The LiFE method requires fascicles in an optimized connectome to contribute to the diffusion prediction by assigning non-zero weights to successful fascicles. Because of this, lesioning fascicles from the model (by setting their weights to zero) increases the prediction error, r.m.s. More specifically, if a set of fascicles, *F*, passes through the set of voxels *V*_*F*_, their path-neighborhood, *P*_*F*_, is defined as all fascicles passing through *V*_*F*_ excluded *F*. The full signal prediction in *V*_*F*_ depends on *F* ∪ *P*_***F***_. The lesioned model instead, predicts the signal in *V*_*F*_ only using *P*_*F*_. The two models of the signal in *V*_*F*_, the lesioned (*P*_*F*_) and unlesioned (*F* ∪ *P*_***F***_) model generate two distributions of r.m.s. error among voxels in *V*_*F*_. These two distributions can be compared using various measures to establish the statistical evidence for given the data^52^.

**Figure 4:**
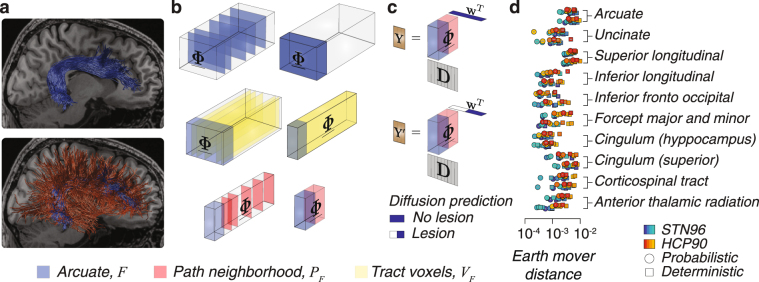
Virtual lesion of white matter tracts using the tensor encoding framework. (**a**) Anatomical representation of the arcuate fasciculus and its path-neighborhood, blue and red respectively. (**b**) Identification of the arcuate fasciculus and its path-neighborhood. **Top**. Arcuate fascicles encoded as frontal slices collated by a permutation (*F*, blue). **Middle**. Ensemble of all voxels touched by the arcuate (lateral tensor slices, yellow) collated by a permutation. **Bottom**. The path-neighborhood (*P*_*F*_, red) contained in the non-empty frontal slices of *V*_*F*_. (**c**) The virtual lesion using the encoding framework. **Top**. Diffusion prediction (**Y**) in the arcuate voxels by the arcuate and its path-neighborhood. **Bottom**. Diffusion prediction (**Y′**) associated to *P*_*F*_, (arcuate fasciculus weights are set to zero, white). (**d**) Statistical evidence for twenty human major white matter tracts^42^ established using the sparse tensor encoding framework. Error bars show ±1 s.e.m.

To date, the virtual lesion method has been employed to establish the statistical evidence for brain tracts and connections^37,39,62,79^. The operations necessary to perform virtual lesions using data represented directly in the brain natural anatomical space require multiple mappings between fascicles coordinates, voxel indices and the corresponding entries in the LiFE model (matrix **M** columns and associated weights). The computational complexity of these operations becomes trivial after encoding connectomes in the multidimensional framework. We show a visualization of the virtual lesion of the right arcuate fasciculus in a single individual (Fig. 4a,b). Given the arcuate fasciculus, *F* (Fig. 4a,b, blue), the identification of *V*_*F*_ and *P*_*F*_, can be achieved in a computationally efficient way using the encoding framework. *V*_*F*_ is the set of lateral slices with non-zero entries within the subtensor identified by *F* (Fig. 4b, yellow) and *P*_*F*_ is the set of fascicles (frontal slices) not in *F* but touching *V*_*F*_ (Fig. 4b, red). Computing the signal prediction with and without lesion is then reduced to evaluate the sparse tensor decomposition and consider the tract weights zero (with lesion) or non-zero (without lesion), as shown in Fig. 4c.

Figure 4d and Supplementary Fig. 4 shows the statistical strength of evidence for twenty major human white matter computed with 19,200 virtual lesions (in all connectomes in Fig. 3) measured as the earth mover distance^52,80^ and strength of evidence^52^. These results are important because they reproduce previous findings^52^ and show large scale reliability of the *in vivo* statistical evidence of major human white matter tracts validated *post mortem* ^41,42^.

### Fourth application: Estimates of white matter geometrical organization

Clarifying the geometrical organization of the brain white matter is emerging as an important opportunity given recent improvements in both, measurement and mapping methods^30,31,57,81–83^. Hereafter, we utilize the encoding framework and 160 statistically validated connectomes to quantify the distribution of angles between white matter fascicles associated with pairs of white matter tracts or between tracts and their path-neighborhood^57–59^.

**Figure 5:**
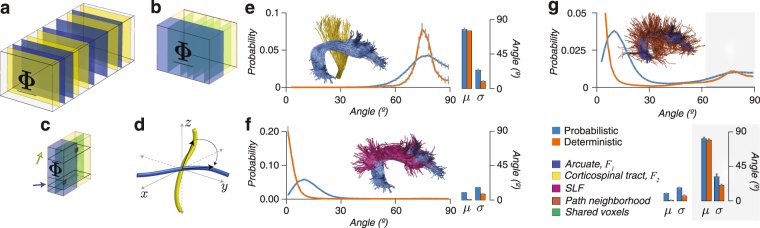
Quantifying variability of estimates for angles of incidence between fascicles in the human white matter. (**a**) Arcuate (Arc, blue) and corticospinal tract (CST, yellow) fascicles identified in frontal slices of $\underline{{\boldsymbol{\Phi }}}$. (**b**) Voxels shared between Arc and CST located by finding lateral slices in $\underline{{\boldsymbol{\Phi }}}$ (green) with non-zero entries in the yellow and blue subtensors. (**c**) Measurement of the angle of incidence in the voxels shared by Arc and CST (green). Angles are determined by finding the indices in the first dimension of $\underline{{\boldsymbol{\Phi }}}$ (1st mode). (**d**) Depiction of angles being computed in brain space. (**e**) Distribution of crossing angles between Arc and CST. (**f**) Distribution of angles incidence between Arc and SLF. (**g**) Distribution of crossing angles between Arc and its neighborhood. Angles computed on Probabilistic (blue) and Deterministic (orange) connectomes (*L*_*max*_ = 10, STN and HCP3T). Analyses based only on fascicles with positive weight. Histograms show mean across subjects (*n* = 8). Bar plots show peak angle (μ) and width-at-half height (*σ*). Error bars ±1 standard error of the mean, s.e.m, across subjects (*n* = 8).

The corticospinal tract (CST), arcuate fasciculus (Arc) and superior lateral fasciculus (SLF) were segmented in the right and left hemispheres of 160 connectomes estimated using either probabilistic or deterministic tractography in eight brains (STN *n* = 4; HCP3T *n* = 4, *L*_*max*_ = 10, ten repeated tracking per brain) and standard atlases^42,74^. Angles between pairs of fascicles within a voxel were estimated by operating on the connectome encoding framework (Fig. 5a–d). We performed three experiments to establish the dependence of fascicle angles on the tracking method and measured the distribution of angles between fascicles in tracts and neighborhoods. We measured: (a) Crossing angles between fascicles in the Arc and CST at voxels of overlap between the tracts. These fascicles were expected to cross with non-zero degree angle. (b) Angles between fascicles in the Arc and SFL. These fascicles were expected to bypass each other with expected angle near zero degrees. (c) Angles between the Arc and its path-neighborhood. The expected angle of crossing between tracts and path neighborhoods has generated important debates^57–59,81^.

We performed three experiments to measure the dependence of angles between white matter fascicles as function of different tracking methods. In the first experiment, we computed pairwise angles between fascicles associated with either of two tracts, *F*_1_ and *F*_2_, the Arc and CST respectively. We began by identifying the fascicles associated with tracts using the frontal slices of $\underline{{\boldsymbol{\Phi }}}$ (3_rd_ mode; Fig. 5a). *F*_1_ and *F*_2_ identify two subtensors, Fig. 5b, blue and yellow respectively. Voxels containing both *F*_1_ and *F*_2_ were selected by finding the lateral slices of $\underline{{\boldsymbol{\Phi }}}$ with non-zero entries in both subtensors (Fig. 5b, green slices, 2nd mode). Finally, we computed all pairwise angles between fascicles in *F*_1_ and *F*_2_ by identifying the atoms (indices in 1st mode) corresponding to the non-zero entries in those lateral slices of $\underline{{\boldsymbol{\Phi }}}$ (Fig. 5c,d).

Using the operations described above, we collected distributions of crossing angles, and computed peak distribution (*μ*) as well as width-at-half-max (*σ*, Fig. 5e). Importantly, we computed approximately 76,000,000 crossing-angles using fascicles validated statistically (fascicles with positive LiFE weights). Crossing angles distributions between Arc and CST peaked approximated at 75° and 78° for deterministic and probabilistic connectomes, respectively (*μ*, Fig. 5e). The measured *σ* was almost three-fold smaller for deterministic than probabilistic connectomes, 9° and 24°, respectively. These results must be put into context by considering the difference in quality of fit of the two connectomes; where probabilistic connectomes on average have a 4.4% lower error (s.d. 1.4%) and 16.2% higher number of supported fascicles (s.d. 1.1%) than deterministic ones (see Fig. 3a, datasets STN and HCP3T). Suplementary Fig. 5a shows the same analyses repeated with a different pair of tracts, the CST and SLF. Results are similar for these tracts with distribution peaking (*μ*) approximately at 78.1° and 86.4° for deterministic and probabilistic connectomes, respectively. Measured was almost two-fold smaller for deterministic than probabilistic connectomes, 17.1° and 31.5°, respectively.

In a second experiment, we measured *μ* and *σ* for the distribution of angles between fascicles within two tracts travelling approximately parallel across the axial plane of the human brain; the Arc and SLF (Fig. 5f). We computed angles distributions for both, probabilistic and deterministic connectomes. The peak distribution (*μ*) was approximately 0° and 15° for deterministic and probabilistic connectomes, respectively. The estimated *σ* were 8.1° and 16.6°, respectively, a 2x increase in variability.

In a final experiment we estimated the distribution of angles between fascicles in a tract, Arc, and its path neighborhood as function of tractography algorithm. Estimates of crossing angles between white matter tracts and path-neighborhoods have been debated^57–59^. We report *μ* and *σ* for crossing angles between the Arc and its path neighborhood using 8 subjects on STN and HCP3T data sets with probabilistic and deterministic (*L*_*max*_ = 10) tracking methods. For each subject, we identified the Arc and its path-neighborhood by using tensorial operations similar to the ones described in Fig. 5a–d. Results show characteristic bimodal distributions (Fig. 5g). A majority of the path-neighborhood fascicles show angles between 0° and 20° with tract fascicles (*μ*, 9° and 0° for probabilistic and deterministic tracking, respectively) and around 80° (*μ*, 81° and 80° for probabilistic and deterministic tracking, respectively). The estimated *σ* for *μ* peaking at around 80° were 20.5° and 31.7° for deterministic and probabilistic connectomes, respectively, a 1.5x increase in variability.

Considering that probabilistic connectomes predict the diffusion measurement better than deterministic ones, these results demonstrate substantial variability in the estimates of crossing angles that can be obtained using neuroimaging methods and that the estimates will depend on the data and analysis methods^57–59^. This result shows a degree of variability of the estimates consistent with recent reports^81,84^.

## Discussion

We presented a connectome encoding framework that provides investigators with an integrated multidimensional relationship between connectome nodes, edges and the associated measurements. We showed the utility of the encoding framework with four applications.

The recent increase in availability, quantity and quality of neuroimaging data and mapping methods poses new opportunities as well as challenges for mapping the human connectome^6–23,54,85^. Technological advances in dMRI data acquisition have permitted reduction of measurement time by factors up to 8-fold^86–88^ and increase in spatial resolution up to 13-fold–when comparing volumetric resolution between clinical and high-field dMRI data^55^ (e.g., 2.5 *mm* and 1.05 *mm* linear resolution respectively). Firstly, increased data quality and resolution also means increased size. Secondly, increased availability and diversity of data accompanied by the established variability in results from tractography, makes it difficult to identify a single tracking algorithm, parameter set or data type valid for every study^52,57,59,62,89,90^. For this reason, developing principled methods for evaluating data quality and tractography routinely in their relation to the connectome estimates has become paramount.

The current practice in mapping connectomes is to choose a single tractography method and data resolution. Yet, multiple reports have been made highlighting many methodological limitations as well as the dependency of results on data and algorithm^89,91–95^. As a result, we now understand that no single tracking method nor data set is likely to solve all problems or provide the ultimate quality. Instead, data and models will need to be improved and carefully evaluated. Routine statistical evaluation of brain connectomes can become standard practice in the process of connectome mapping^31,52,63,64,96^. The proposal is to build predictive models of the measured dMRI signal from the structure of brain connectomes^52,62,96^ and compare the model prediction to the data by using statistical methods such as cross-validation^97^. The statistical evaluation approach complements the work on tractography validation based on either synthetic or post-mortem preparations^90,98,99^. Previous work evaluated model accuracy, namely how well a tractography method predicts independent dMRI measurements^52^. The present work advances by measuring model precision, how similar connectome estimates are when using a single tractography method repeatedly.

Multidimensional decomposition methods have been used to help investigators make sense of large multimodal datasets^49,100^. Yet to date these methods have found only a few applications in neuroscience, such as performing multi-subjects, clustering and electroencephalography analyses^48,101–106^. Generally, decomposition methods have been used to find compact representations of complex data by estimating the combination of a limited number of common meaningful factors that best fit the data^50,100,107^. We propose a new application that instead of using the decomposition to estimate latent factors, it encodes the structure of the problem explicitly. This innovative application in neuroscience can open new avenues of investigation in mapping brain and behavior using multivariate methods^108,109^ and to allow improving future generations of models of connectomics, tractography evaluation and microstructure^52,62–64^. Improving these models will allow going beyond current limitations of the state of the art methods^92^. For example, extensions of the proposed framework would allow building more complex relationships between connectome matrices, edges and nodes without the loss of information of dMRI data and fascicles properties inherent to current methods for connectomics^4^.

The field of network neuroscience^4^ and the study of white matter^31,110,111^ are striving to improve methods for mapping connectomes using modern large-scale data sets from living human brains. Our results show that connectome evaluation can be applied on such data sets with thousands of brain. In addition, the results show a profound effect of dMRI data spatial resolution on the number of brain connections that can be mapped. The effect of spatial resolution goes even beyond that of directional resolution that is lower for the HCP7T than the HCP3T data set used here^55^. This is particularly important because of its implications in guiding future study design.

Advances in frameworks to integrate computations on fascicles, brain areas as well as dMRI data, can profoundly improve efforts in clarifying the properties of human brain macroscopic connectivity^1,29,30,108^ and white matter microstructure^112–117^. Data representation frameworks such as the one proposed here have the potential to become fundamental in advancing the application of machine learning algorithms to mapping the functional, structural properties of the human connectome to capture brain individuality and variability in health and disease^21,35,73,81,118–122^. To contribute advancing scientific understanding and reproducibility, we provide an open source implementation of the encoding method and files to reproduce figures at http://www.github.com/brain-life/encode.

## Methods

### Diffusion-weighted MRI datasets

We use diffusion-weighted Magnetic Resonance Imaging data (dMRI) from three publicly available sources^22,51,52,54,55^. Dataset are available online at http://purl.stanford.edu/rt034xr8593, http://purl.stanford.edu/ng782rw8378 and https://www.humanconnectome.org/data/.

#### Stanford datasets

*STN*, 96 *gradient directions*, 1.5 *mm isotropic resolution*. dMRI dataset were collected in five males subjects (age 37–39) at the Stanford Center for Cognitive and Neurobiological Imaging using a 3 T General Electric Discovery 750 (General Electric Healthcare) equipped with a 32-channel head coil (Nova Medical). dMRI datasets with whole-brain volume coverage were acquired using a dual-spin echo diffusion-weighted sequence. Water-proton diffusion was measured using 96 directions chosen using the electrostatic repulsion algorithm^123^. Diffusion-weighting gradient strength was set to 2,000 *s*/*mm* ^2^ (*TE* = 96.8 *ms*). Data were acquired at 1.5 *mm* isotropic spatial resolution. Individual datasets were acquired twice and averaged in k-space (*NEX* = 2). Ten non-diffusion-weighted (*b* = 0) images were acquired at the beginning of each scan. Data acquisition and preprocessing steps are described in^52^.

*STN150*, 150 *gradient directions*, 2.0 *mm isotropic resolution*. dMRI data were acquired in one subject using 150 directions, 2 *mm* isotropic spatial resolution and *b* value of 2,000 *s*/*mm* ^2^ (*TE* = 83.1,93.6, and 106.9 *mm*).

Data acquisition and preprocessing steps are described in^52^.

#### Human Connectome Project datasets

*HCP3T*, 90 *gradient directions*, 1.25 *mm isotropic resolution*. Data of four subjects, part of the Human Connectome Project^54^, acquired using a Siemens 3 T “Connectome” scanner were used. Measurements from the 2,000 *s*/*mm* ^2^ shell were extracted from the original dataset and used for all analyses. Processing methods are described in^22^.

*HCP7T*, 60 *gradient directions*, 1.05 *mm isotropic resolution*. Five subjects part of the Human Connectome 7-Tesla (7 T) dataset were used. Data were collected a Siemens 7 T scanner^55^. Measurements from the 2,000 *s*/*mm* ^2^ shell were extracted from the original data and were used for further analyses.

### Whole-brain connectomes generation

Tractography was performed using the MRtrix 0.2 toolbox^67^. White-matter tissue was identified from the cortical segmentation performed on the T1-weighted images and resampled at the resolution of the dMRI data. Only white-matter voxels were used to seed fiber tracking. We used three tracking methods: (i) tensor-based deterministic tracking^67–69^, (ii) CSD-based deterministic tracking^66,67^, and (iiI) CSD-based probabilistic tracking^66,67,124,125^. Maximum harmonic orders (*L*_*max*_) of 2, 4, 6, 8, 10 and 12 were used as long as the number of directions is larger than the number of parameters *N*_p_ = 0.5(*L*_*max*_ + 1)(*L*_*max*_ + 2)^66,126^. The following parameter values were used for all tracking: step size: 0.2 *mm*; minimum radius of curvature, 1 *mm*; maximum length, 200 *mm*; minimum length, 10 *mm*; and the fibers orientation distribution function (*f*_*ODF*_) amplitude cutoff, was set to 0.1.

We created 10 candidate whole-brain connectomes by repeating tracking using 500,000 fascicles in each individual brain dataset (fourteen), tractography method (three) and parameter *L*_*max*_ (six).

A total number of 1,490 connectomes were generated in this work. For each connectome, fascicles of the twenty major human were identified using Automatic Fiber Quantification - AFQ^74^.

### The Linear Fascicle Evaluation (LiFE) method

Here we introduce the linear model used in^52^ to predict diffusion signals based on a multi-compartment voxel model^127,128^. We refer to Supplementary section 1.2 for an introduction to magnetic resonance diffusion signals.

For a given sensitization strength *b* and gradient direction ***θ***, the diffusion signal *S*(**θ**,*v*) measured at a location within a brain (voxel *v*) can be estimated by using the following Equation:

1 $$S({\boldsymbol{\theta }},v)\approx {S}_{0}(v)({w}_{0}{e}^{-{A}_{0}}+\sum _{f\in v}{w}_{f}{e}^{-b{{\boldsymbol{\theta }}}^{T}{{\bf{Q}}}_{f,v}{\boldsymbol{\theta }}}),$$

where *f* is the index of the candidate white-matter fascicles within the voxel, *S*(**θ**,*v*) is the diffusion-weighted signal, *S*_0_(*v*) is the non diffusion-weighted signal (*b* = 0), *A*_0_ is the isotropic apparent diffusion (diffusion in all directions) and **Q**_*f,v*_ is the diffusion tensor matrix (see Supplementary section 1.2).

LiFE predicts the demeaned diffusion signal defined as $\bar{S}({\boldsymbol{\theta }},v)=S({\boldsymbol{\theta }},v)-{I}_{v}$, where ${I}_{v}=\frac{1}{{N}_{{\boldsymbol{\theta }}}}{\sum }_{{\boldsymbol{\theta }}}S({\boldsymbol{\theta }},v)$ is the mean and *N*_θ_ is the number of gradient directions^51,52^. Using this definition and Equation (1) we arrive at:

2 $$\bar{S}({\boldsymbol{\theta }},v)\approx \sum _{f\in v}{w}_{f}{S}_{0}(v){O}_{f}({\boldsymbol{\theta }},v),$$

where and *O*_*f*_(**θ**,*v*) is the orientation distribution function specific to each fascicle, i.e. the anisotropic modulation of the diffusion signal around its mean and it is defined as follows:

3 $${O}_{f}({\boldsymbol{\theta }},v)={e}^{-b{{\boldsymbol{\theta }}}^{T}{{\bf{Q}}}_{f,v}{\boldsymbol{\theta }}}-\frac{1}{{N}_{{\boldsymbol{\theta }}}}\sum _{{\boldsymbol{\theta }}}{e}^{-b{{\boldsymbol{\theta }}}^{T}{{\bf{Q}}}_{f,v}{\boldsymbol{\theta }}}.$$

The right-hand side of Equation (2) is the prediction model (see Supplementary Fig. 2a,b). The LiFE model extends from the single voxel to all white-matter voxels in the following way (see Supplementary Fig. 2c):

4 $${\bf{y}}\approx {\bf{M}}{\bf{w}},$$

where ${\bf{y}}\,\in \,{{\rm{R}}}^{{N}_{{\boldsymbol{\theta }}}{N}_{v}}$ is a vector containing the demeaned signal for all white-matter voxels *v* and across all gradient directions **θ**, i.e. ${y}_{i}=\bar{S}({{\boldsymbol{\theta }}}_{i},{v}_{i})$. The matrix ${\bf{M}}\in {{\rm{R}}}^{{N}_{{\boldsymbol{\theta }}}{N}_{v}\times {N}_{f}}$ contains at column *f* the signal contribution given by fascicle *f* at all voxels across all gradient directions, i.e., **M**(*i*,*f*) = *S*_0_(*v*_*i*_)*O*_*f*_(**θ**_*i*_), and ${\bf{w}}\in {{\rm{R}}}^{{N}_{f}}$ contains the weights for each fascicle in the connectome.

The vector of weights **w** in Equation (4) and Supplementary Fig. 2c is computed by solving a convex optimization problem^52,63^. More specifically we solve a non-negative least-square (NNLS) problem, defined as follows:

5 $$\mathop{{\rm{\min }}}\limits_{{\bf{w}}}(\frac{1}{2}{\Vert {\bf{y}}-{\bf{M}}{\bf{w}}\Vert }^{2})\,\,{\rm{subject}}\,{\rm{to}}\,{w}_{f}\ge \mathrm{0,}\forall f\mathrm{.}$$

Commonly, the size of the matrix **M** is very large (around 30 *GB* or 40 *GB* for the datasets used here, see Fig. 2d). Because of this reason, we use NNLS algorithms suitable for large scale problems, such as the BB-NNLS developed in^129^.

#### Connectome model prediction error

LiFE predicts the measured (demeaned) diffusion signal using the right-hand side of Equation (2). Thus, we can assess the ability of LiFE to model the measured diffusion signal by computing the prediction error in each white-matter voxel. In order to make errors relatively independent of scanner parameters, we compute them on the relative diffusion signal (also referred to as diffusion attenuation), defined as follows:

6 $${\bar{S}}_{r}({\boldsymbol{\theta }},v)=\bar{S}({\boldsymbol{\theta }},v)/{S}_{0}(v\mathrm{).}$$

The root mean squared (r.m.s) error in voxel *v* is defined as follows:

7 $${e}_{rms}(v)=\sqrt{\frac{1}{{N}_{\theta }}{\sum _{{\boldsymbol{\theta }}}({\bar{S}}_{r}({\boldsymbol{\theta }},v)-\sum _{f\in v}{w}_{f}{O}_{f}({\boldsymbol{\theta }},v))}^{2}}\mathrm{.}$$

The r.m.s error (Equation 7) can be used to compare alternative connectome models. A global r.m.s error ${\bar{e}}_{rms}$ can be computed by averaging *e*_*rms*_(*v*) over all voxels:

8 $${\bar{e}}_{rms}=\frac{1}{{N}_{v}}\sum _{v}{e}_{rms}(v)\mathrm{.}$$

#### LiFE models comparison

We compare a LiFE_M_ model matrix **M** (see Equation 4) and its approximated version $\hat{{\bf{M}}}$ using the relative error:

9 $${e}_{{\bf{M}}}={\Vert {\bf{M}}-\hat{{\bf{M}}}\Vert }_{F}/{\Vert {\bf{M}}\Vert }_{F},$$

where ${\Vert {\bf{M}}\Vert }_{F}=\sqrt{{\sum }_{i,j}{{\bf{M}}}^{2}(i,j)}$ is the Frobenius matrix norm.

Similarly, we compare a vector of LiFE_M_ weights **w** and its approximated version $\hat{{\bf{w}}}$ using the relative error defined as follows:

10 $${e}_{{\bf{w}}}=\Vert {\bf{w}}-\hat{{\bf{w}}}\Vert /\Vert {\bf{w}}\Vert ,$$

where $\Vert {\bf{w}}\Vert =\sqrt{\sum _{f}{w}_{f}^{2}}$ is the Euclidean vector-norm.

## Electronic supplementary material

Supplementary Text and Figures
