# Supplementary material for: Multidimensional encoding of brain connectomes
Source: Sci Rep. 2017 Sep 13;7:11491. doi: 10.1038/s41598-017-09250-w (PMC5597641; doi:10.1038/s41598-017-09250-w)
Supplement: Supplementary file 1 — Supplementary Text and Figures [file 41598_2017_9250_MOESM1_ESM.pdf]

Multidimensional encoding of brain connectomes

Cesar F. Caiafa[1,2,3] **and Franco Pestilli**[1]

[1]Department of Psychological and Brain Sciences, Programs in Neuroscience and Cognitive Science, Indiana Network Science Institute, Indiana University, Bloomington, IN, 47405, USA
[2]Instituto Argentino de Radioastronomía (IAR), CONICET, CCT - La Plata, Villa Elisa, 1894, ARGENTINA
[3]Facultad de Ingeniería - Departamento de Computación - UBA, Buenos Aires, C1063ACV, ARGENTINA.

SUPPLEMENTARY INFORMATION

Contents

1 Supplementary Methods **2**
1.1 Multidimensional arrays notation and definitions . . . 2
1.2 Mathematical Modeling of dMRI signals . . . 5
2 Supplementary Results **5**
2.1 Tensor decomposition of the Linear Fascicle Evaluation model . . . 5
2.2 Comparison of the weights estimated by LiFE_M and LiFE_T . . . 6
2.3 Encoding a connectome into LiFE_T . . . 6
Comparison between the multidimensional encoding framework with the standard fascicles representation
2.4 Fitting the LiFE_T model . . . 7
Computing $\mathbf{y} = \mathbf{M}\mathbf{w}$ • Computing $\mathbf{w} = \mathbf{M}^T\mathbf{y}$
2.5 Analysis of the LiFE_T model accuracy . . . 9
2.6 Analysis of the LiFE_T model compression factor . . . 10
References **15**

1 Supplementary Methods

1.1 Multidimensional arrays notation and definitions

Multidimensional arrays (tensors) generalize vectors (1D array) and matrices (2D array) to arrays of higher dimensions, three or more. Such arrays can be used to perform multidimensional factor analysis and decomposition and are of interest to many scientific disciplines[1,2].

Below, we introduce basic concepts and notation (we refer the reader to **Supplementary Table 1**).

Vectors, matrices and tensors. Vectors and matrices are denoted using boldface lower- and upper-case letters, respectively. For example $\mathbf{x} \in \mathbb{R}^I$ and $\mathbf{X} \in \mathbb{R}^{I\times J}$ represent a vector and a matrix, respectively. A tensor $\underline{\mathbf{X}} \in \mathbb{R}^{I\times J\times K}$ is a 3D array of real numbers whose elements (i, j, k) are referred to as $\mathbf{X}(i, j, k)$ or x_{ijk}. The individual dimensions of a tensor are referred to as modes (1st mode, 2nd mode, and so on).

Tensor slices and mode-n vectors. Slices are used to address a tensor along a single dimension and are obtained by fixing the index of one dimension of the tensor while letting the other indices vary. For example, in a 3D tensor $\underline{\mathbf{X}} \in \mathbb{R}^{I\times J\times K}$, we identify horizontal (i), lateral (j) and frontal (k) slices by holding fixed the corresponding index of each array dimension (see **Supplementary Fig. 1a**-top). Tensors can be also addressed in any dimension by means of mode-n vectors. These vectors are obtained by holding all indices fixed except one (**Supplementary Fig. 1a**-bottom).

Subtensors and tensor unfolding. A subset of indices in any mode identifies a volume also referred as to a subtensor. For example, in **Fig. 1d**, we identify a volume by collecting slices in the 3rd mode. In addition, a tensor can be converted into a matrix by re-arranging its entries (unfolding). The mode-n unfolded matrix, denoted by $\mathbf{X}_{(n)} \in \mathbb{R}^{I_n\times \bar{I}_n}$, where $\bar{I}_n = \prod_{m\neq n} I_m$ and whose entry at row i_n and column $(i_1-1)I_2\cdots I_{n-1}I_{n+1}\cdots I_N + \cdots + (i_{N-1}-1)I_N + i_N$ is equal to $x_{i_1 i_2 \ldots i_N}$. For example, mode-2 unfolding builds the matrix $\mathbf{X}_{(2)}$ where its columns are the mode-2 vectors of the tensor and the rows are vectorized versions of the lateral slices, i.e. spanning dimensions with indices i and k (see **Supplementary Fig. 1b**).

Tensor by matrix product. By generalization of matrix multiplication, a tensor can be multiplied by a matrix in a specific mode, only if their size matches. Given a tensor $\underline{\mathbf{X}} \in \mathbb{R}^{I_1\times I_2\cdots\times I_N}$ and a matrix $\mathbf{A} \in \mathbb{R}^{J\times I_n}$, the mode-$n$ product

$$\underline{\mathbf{Y}} = \underline{\mathbf{X}} \times_n \mathbf{A} \in \mathbb{R}^{I_1\times\cdots\times I_{n-1}\times J\times I_{n+1}\cdots\times I_N} \tag{11}$$

is defined by: $y_{i_1\cdots i_{n-1} j i_{n+1}\cdots i_N} = \sum_{i_n=1}^{I_n} x_{i_1\cdots i_n\cdots i_N} a_{j i_n}$, with $i_k = 1,2,\ldots,I_k$ $(k \neq n)$ and $j = 1,2,\ldots,J$. **Supplementary Fig. 1c** illustrates a 3D tensor by matrix product operation (2nd mode, $\underline{\mathbf{Y}} = \underline{\mathbf{X}} \times_2 \mathbf{A}$).

Tucker decomposition. Low-rank matrix approximation can be generalized to tensors by Tucker decomposition[3]. For example, $\underline{\mathbf{X}} \in \mathbb{R}^{I_1\times I_2\times I_3}$, can be approximated by:

$$\underline{\mathbf{X}} \approx \underline{\mathbf{G}} \times_1 \mathbf{A}_1 \times_2 \mathbf{A}_2 \times_3 \mathbf{A}_3, \tag{12}$$

where $\times_n$ is the mode-n tensor-by-matrix product. $\underline{\mathbf{G}} \in \mathbb{R}^{R_1\times R_2\times R_3}$ is the *core tensor* and $\mathbf{A}_n \in \mathbb{R}^{I_n\times R_n}$ are *factor matrices*. Such a decomposition guarantees data compression when the core tensor is much smaller than the original, i.e. $R_n \ll I_n$ (see **Supplementary Fig.1d**-top).

Sparse Decomposition: Tensors can be approximated also by sparse decomposition[4,5]. In this case, compression can be achieved independently of the size of $\underline{\mathbf{G}}$ as long as sparsity is sufficiently high (see **Supplementary Fig.1d**-bottom).

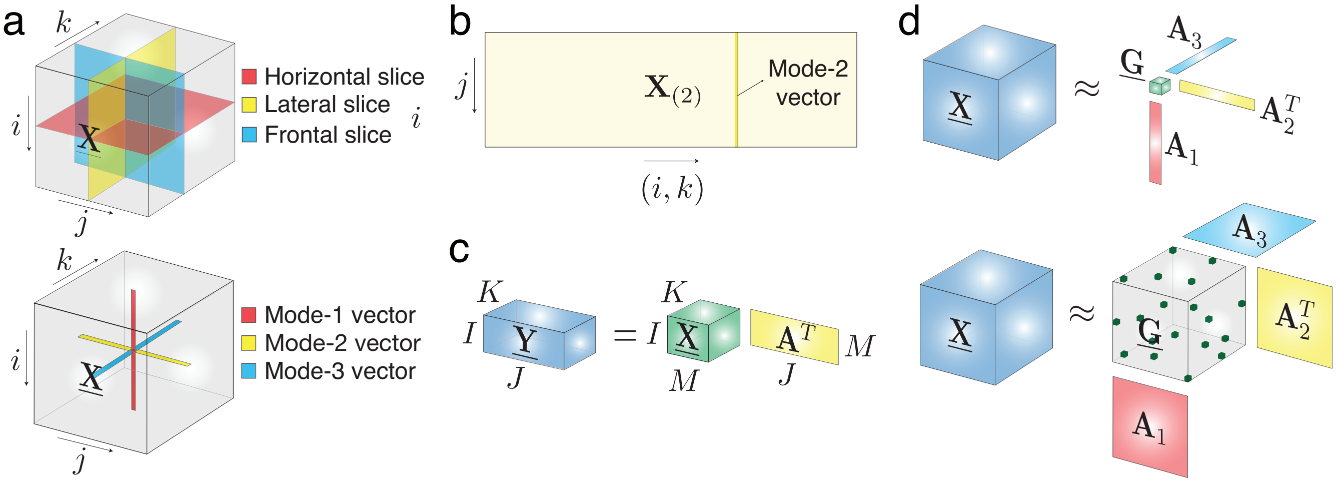

Supplementary Fig. 1. **(a)** Examples of frontal (light blue), lateral (yellow) and horizontal (red) slices of 3D tensor (top), and examples of mode-n vectors (bottom). **(b)** Illustration of the mode-2 unfolded matrix $\mathbf{X}_{(2)}$. **(c)** Tensor-by-matrix product (example of product in mode-2). **(d)** The classical Tucker decomposition (top;[3]) allows representing a 3D tensor $\underline{\mathbf{X}} \in \mathbb{R}^{I_1 \times I_2 \times I_3}$ as the product of a core tensor (green) $\underline{\mathbf{G}} \in \mathbb{R}^{R_1 \times R_2 \times R_3}$ by factor matrices $\mathbf{A}_n \in \mathbb{R}^{I_n \times R_n}$ (red, yellow and light blue). Data compression is achieved by considering very small (dense) core tensors $\underline{\mathbf{G}}$, meaning that $R_n \ll I_n$. The sparse Tucker Decomposition (bottom;[4]). The core tensor $\underline{\mathbf{G}}$ is large but sparse. Data compression is achieved because of the sparsity of the core tensor. See **Supplementary Table 1** for additional information about notation and mathematical definitions.

Table 1. Mathematical notation and definitions for multidimensional arrays (tensors) and their decomposition.

$\underline{\mathbf{X}}$, $\mathbf{A}$, $\mathbf{w}$, b	A tensor, a matrix, a vector and a scalar
$x_{i_1 i_2 \dots i_N}$, a_{ij}, w_i	Entries of a tensor, a matrix and a vector
$\underline{\mathbf{X}}(:,j,k)$, $\underline{\mathbf{X}}(i,:,k)$, $\underline{\mathbf{X}}(i,j,:)$	Mode-1, mode-2 and mode-3 vectors are obtained by fixing all but one index
$\underline{\mathbf{X}}(i,:,:)$, $\underline{\mathbf{X}}(:,j,:)$, $\underline{\mathbf{X}}(:,:,k)$	Horizontal, lateral and frontal slices are obtained by fixing all but two indices
$\mathbf{X}_{(n)} \in \mathbb{R}^{I_n \times I_1 I_2 \cdots I_{n-1} I_{n+1} \cdots I_N}$	Mode-n unfolding of tensor $\underline{\mathbf{X}} \in \mathbb{R}^{I_1 \times I_2 \times \cdots \times I_N}$ whose entry at row i_n and column $(i_1 - 1) I_2 \cdots I_{n-1} I_{n+1} \cdots I_N + \cdots + (i_{N-1} - 1) I_N + i_N$ is equal to $x_{i_1 i_2 \dots i_N}$
$\underline{\mathbf{Y}} = \underline{\mathbf{X}} \times_n \mathbf{A} \in \mathbb{R}^{I_1 \times \cdots \times I_{n-1} \times J \times I_{n+1} \cdots \times I_N}$	tensor by matrix product (in mode-n) where $y_{i_1 \cdots i_{n-1} j i_{n+1} \cdots i_N} = \sum_{i_n=1}^{I_n} x_{i_1 \cdots i_n \cdots i_N} a_{j i_n}$
$\underline{\mathbf{X}} \approx \underline{\mathbf{G}} \times_1 \mathbf{A}_1 \times_2 \mathbf{A}_2 \times_3 \mathbf{A}_3$	Tucker decomposition: a 3D tensor $\underline{\mathbf{X}} \in \mathbb{R}^{I_1 \times I_2 \times I_3}$ is represented as the product of a core array $\underline{\mathbf{G}} \in \mathbb{R}^{R_1 \times R_2 \times R_3}$ by factor matrices $\mathbf{A}_n \in \mathbb{R}^{I_n \times R_n}$
$\mathbf{x} = vec(\underline{\mathbf{X}}) \in \mathbb{R}^{I_1 I_2 \cdots I_N}$	Vectorization of tensor $\underline{\mathbf{X}} \in \mathbb{R}^{I_1 \times I_2 \times \cdots \times I_N}$ with the entry at position $i_1 + \sum_{k=2}^{N} [(i_k - 1) I_1 I_2 \cdots I_{k-1}]$ equal to $x_{i_1 i_2 \cdots i_N}$

1.2 Mathematical Modeling of dMRI signals

Measured dMRI signals depend on the combination of multiple cellular compartments within the brain tissue[6] (e.g., neurons, astrocytes and oligodendrocytes). The dMRI signal is generally modeled as the linear combination of two components. One component describes the directional diffusion signal and is presumably related primarily to the direction of the neuronal axons wrapped in myelin sheaths (white matter). This signal is often referred to as *anisotropic diffusion*. The other component describes *isotropic diffusion* (non-directional) and is presumably related to the combination of signals originating from the rest of the cellular bodies within the brain tissue. Below we introduce the equations we used to model the dMRI signal in relation to these compartments.

dMRI measurements are collected with and without a diffusion sensitization magnetic gradient. Such gradient allows the dMR image intensity to vary depending on water diffusion along a single direction. Generally multiple dMR images are collected for each brain location by varying this diffusion-sensitization gradient (i.e., by sequentially orienting the gradient along several gradient directions). The measured signal depends on a combination of parameters such as diffusion gradient strength and duration. Below we denote the diffusion sensitization gradient strength with the scalar b and direction with the unit-norm vector $\boldsymbol{\theta} \in \mathbb{R}^3$.

For a given sensitization strength b and diffusion direction $\boldsymbol{\theta}$, the measured dMRI signal at each location within a brain (voxel v) can be computed using the following equation[7,8]:

$$S(\boldsymbol{\theta}, v) \approx \mathbf{w}_0 S_0(v) e^{-A_0} + \sum_{f \in v} \mathbf{w}_f S_0(v) e^{-b\boldsymbol{\theta}^T \mathbf{Q}_{f,v} \boldsymbol{\theta}}, \tag{13}$$

where f is the index of the candidate white-matter fascicles in the voxel, $S_0(v)$ is the non diffusion-weighted signal in voxel v and A_0 is the isotropic apparent diffusion (diffusion in all directions). The value $\boldsymbol{\theta}^T \mathbf{Q}_{f,v} \boldsymbol{\theta} > 0$ gives us the apparent diffusion at direction $\boldsymbol{\theta}$ generated by fascicle f. $\mathbf{Q}_{f,v} \in \mathbb{R}^{3\times3}$ is a symmetric and positive-definite matrix called diffusion tensor[9]. The diffusion tensor $\mathbf{Q}_{f,v}$ allows a compact representation of the diffusion signal measured with dMRI. Usually, $\mathbf{Q}_{f,v}$ is represented by an 3D-ellipsoid as shown in **Supplementary Fig. 2a**, which can be mathematically defined with the equation:

$$\mathbf{Q}_{f,v} = \begin{bmatrix} \mathbf{u}_1 & \mathbf{u}_2 & \mathbf{u}_3 \end{bmatrix} \begin{bmatrix} s_a & 0 & 0 \\ 0 & s_{r_1} & 0 \\ 0 & 0 & s_{r_2} \end{bmatrix} \begin{bmatrix} \mathbf{u}_1 \\ \mathbf{u}_2 \\ \mathbf{u}_3 \end{bmatrix}, \tag{14}$$

where $\mathbf{u}_n \in \mathbb{R}^{3\times1}$ are the unit-norm orthogonal vectors that correspond to the semi-axes of the diffusion tensor ellipsoid, and s_a, s_{r_1}, s_{r_2} define the axial and radial diffusivity of the tensor, respectively. In the simplest version of the model, $s_a = 1$ and $s_{r_1} = s_{r_2} = 0$, which means that diffusion is restricted to the main axis direction (stick model)[8,10].

2 Supplementary Results

2.1 Tensor decomposition of the Linear Fascicle Evaluation model

The LiFE[10] method predicts the demeaned diffusion signal $\mathbf{y} \in \mathbb{R}^{N_{\boldsymbol{\theta}} N_v}$ in all voxels ($v = 1, 2, \ldots, N_v$) and gradient directions ($\boldsymbol{\theta}_1, \boldsymbol{\theta}_2, \ldots, \boldsymbol{\theta}_{N_{\boldsymbol{\theta}}}$) using the following equation (see **Methods** for its derivation). Each column in matrix $\mathbf{M}$ (**Methods**, equation 4) contains the diffusion signal contribution from a single fascicle at all voxels and gradient directions. Vector $\mathbf{w} \in \mathbb{R}^{N_f}$ contains the weights associated to each fascicle's contribution.

Vector $\mathbf{y}$ and matrix $\mathbf{M}$ are composed by a vertical concatenation of N_v block vectors $\mathbf{y}_v \in \mathbb{R}^{N_{\boldsymbol{\theta}}}$ and matrices $\mathbf{M}_v \in \mathbb{R}^{N_{\boldsymbol{\theta}} \times N_f}$, where each block corresponds to a particular voxel v (see **Supplementary Fig. 2c**):

$$\begin{bmatrix} \mathbf{y}_1 \\ \mathbf{y}_2 \\ \vdots \\ \mathbf{y}_{N_v} \end{bmatrix} \approx \begin{bmatrix} \mathbf{M}_1 \\ \mathbf{M}_2 \\ \vdots \\ \mathbf{M}_{N_v} \end{bmatrix} \mathbf{w}, \tag{15}$$

Thus, in each voxel we have the following linear model: $\mathbf{y}_v \approx \mathbf{M}_v \mathbf{w}$. Matrix $\mathbf{M}_v$ can be factorized as follows

$$\mathbf{M}_v \approx \hat{\mathbf{M}}_v = \mathbf{D}\boldsymbol{\Phi}_v, \tag{16}$$

where matrix $\mathbf{D} \in \mathbb{R}^{N_{\boldsymbol{\theta}} \times N_a}$ is a dictionary of diffusion predictions whose columns (atoms) correspond to precomputed fascicle orientations, and $\boldsymbol{\Phi}_v \in \mathbb{R}^{N_a \times N_f}$ is a sparse matrix whose non-zero entries ($\boldsymbol{\Phi}_v(a, f)$) indicate the orientation of fascicle f in voxel v approximated by atom a. The dictionary atoms were created by uniformly sampling the azimuth (α) and elevation

(β) of an idealized unit-norm sphere representing the space of putative fascicles directions (**Supplementary Fig. 2f**). More specifically, the entries of the dictionary were computed as follows:

$$\mathbf{D}(\boldsymbol{\theta}, a) = e^{-b\boldsymbol{\theta}^T \mathbf{Q}_a \boldsymbol{\theta}} - \frac{1}{N_\theta} \sum_{\theta} e^{-b\boldsymbol{\theta}^T \mathbf{Q}_a \boldsymbol{\theta}}, \tag{17}$$

where $\mathbf{Q}_a$ is a diffusion tensor that approximates $\mathbf{Q}_{f,v}$. At voxels where a fascicle is straight enough, the diffusion signal is approximated by addressing only one atom (one orientation), however for curved fascicles at a voxel its diffusion signal is approximated by a linear combination of few atoms in the dictionary. However, matrix $\mathbf{\Phi}_v$ follows the constraint

$$\sum_a \mathbf{\Phi}_v(a, f) = S_0(v). \tag{18}$$

By inserting equation (16) into equation (15), and transforming the approximated full matrix $\hat{\mathbf{M}}$ of the original LiFE model[10] into a tensor $\underline{\hat{\mathbf{M}}} \in \mathbb{R}^{N_a \times N_v \times N_f}$ (stacking block matrices $\mathbf{M}_v$ as lateral slices[a]), we demonstrate that the following decomposition holds:

$$\underline{\hat{\mathbf{M}}} = \underline{\mathbf{\Phi}} \times_1 \mathbf{D}, \tag{19}$$

where $\underline{\mathbf{\Phi}} \in \mathbb{R}^{N_a \times N_v \times N_f}$ is obtained by stacking all individual voxels matrices $\mathbf{\Phi}_v$ ($v = 1, 2, \ldots, N_v$) into the lateral slices of $\underline{\mathbf{\Phi}}$.

Finally, using equations (4) and (19), the full LiFE_T model can be written as (**Fig. 2a**):

$$\mathbf{Y} \approx \underline{\mathbf{\Phi}} \times_1 \mathbf{D} \times_3 \mathbf{w}^T, \tag{20}$$

where matrix $\mathbf{Y} \in \mathbb{R}^{N_{\boldsymbol{\theta}} \times N_v}$ is obtained by stacking vectors $\mathbf{y}_v$ ($v = 1, 2, \ldots, N_v$) as columns, $\underline{\mathbf{\Phi}} \in \mathbb{R}^{N_a \times N_v \times N_f}$ is a sparse core tensor and $\mathbf{w} \in \mathbb{R}^N_f$ is the vector of weights (see **Supplementary Fig. 2e**). This model is an example of application of Sparse Tucker Decomposition (see **Methods**,[4]). In the article we refer to this model as LiFE_T (see **Fig. 2a**).

2.2 Comparison of the weights estimated by LiFE_M and LiFE_T

In equation (13), we introduced a measure that quantifies the difference between two weights vectors $\mathbf{w}$ and $\hat{\mathbf{w}}$. Weights vectors are sparse and their entries indicate which fascicles in the connectome contribute (non-zero) or not (zero) to predict the diffusion signal. **Fig. 2c**, bottom panel, shows a global comparison of the vector of weights estimated by LiFE and LiFE_T as defined in equation (13).

Hereafter, we perform additional detailed analyses on the difference between these vectors. The observed error between the vectors $\mathbf{w}$ and $\hat{\mathbf{w}}$ can, in theory, be the result of: (1) each model assigning non-zero weights to a different subset of fascicles; (2) non-zero weights being assigned to the same subset of fascicles but their magnitude differs in the two models; (3) a combination of (1) and (2). This difference is important because it would indicate that either LiFE_M and LiFE_T select very different fascicles (1) or the same fascicles (2). The case in (1) would indicate a potential bias in LiFE_T. We explicitly quantified which one of these three cases contributed to the observed error in the weights. To do this, we defined two subsets of weight-fascicles indices. Those that have non-zero values in both models, *common-fascicles indices* (**Supplementary Fig. 2j**, orange) and those that have non-zero values in one model but not in the other, *different-fascicles indices* (**Supplementary Fig. 2j**, green and blue).

We define the vectors of *common-fascicles* as $\mathbf{w}_c$ and $\hat{\mathbf{w}}_c$, and *different-fascicles* as $\mathbf{w}_d$ and $\hat{\mathbf{w}}_d$ (see **Supplementary Fig. 2j**). We demonstrate that the square of the error of the weights (**Methods**, equation 13) is:

$$e^2_{\mathbf{w}} = e^2_{\mathbf{w}_c} + e^2_{\mathbf{w}_d}, \tag{21}$$

where $e^2_{\mathbf{w}_c} = \frac{\|\mathbf{w}_c - \hat{\mathbf{w}}_c\|^2}{\|\mathbf{w}\|^2}$ and $e^2_{\mathbf{w}_d} = \frac{\|\mathbf{w}_d\|^2}{\|\mathbf{w}\|^2} + \frac{\|\hat{\mathbf{w}}_d\|^2}{\|\mathbf{w}\|^2}$ are the squared errors associated to the *common-* and *different-fascicles*, respectively.

2.3 Encoding a connectome into LiFE_T

Encoding a brain connectome information into the LiFE_T model involves the computation of the dictionary matrix $\mathbf{D}$ and the sparse tensor $\underline{\mathbf{\Phi}}$.

The dictionary matrix $\mathbf{D}$ need to be computed first by fixing the total number of atoms N_a to be used, i.e. by setting the minimum incremental unit $\Delta = \pi/L$ for the spherical coordinates which create a regular grid (see **Supplementary Fig. 2f**). Thus, by increasing the parameter L we increase the resolution which has two main consequences: 1) reduces the

[a] Note that the $\hat{\mathbf{M}}$ is the transposed mode-3 unfolded matrix of tensor $\underline{\hat{\mathbf{M}}}$, i.e $\hat{\mathbf{M}} = \hat{\mathbf{M}}^T_{(3)}$. We use this unfolding operation to compute the LiFE_T model error $\mathbf{e}_\mathbf{M}$, see **Fig. 2c**, top panel.

approximation error (see **section 2.5** and **Supplementary Fig. 2g-i**), and 2) increases the size of the model (see **section 2.6**). We have demonstrated that by using $L = 360$, we obtained a very accurate approximation and a substantial reduction in storage requirements.

By computing the sparse tensor $\underline{\mathbf{\Phi}}$ we encode the information of fascicles in a connectome into our decomposed model. More specifically, the sparse tensor is computed slice by slice (mode-3), i.e. by looking at one fascicle in the connectome at a time. Each fascicle f is composed by a set of nodes connected, so for each one of the nodes we need: 1) to identify the voxel index v in which the node is located, and 2) find the atom index a having the spatial orientation of that fascicle. Finally, for each fascicle node we set a non-zero entry within the sparse tensor $\underline{\mathbf{\Phi}}$ as follows:

$$\underline{\mathbf{\Phi}}(a, v, f) = 1. \tag{22}$$

After encoding all the fascicles nodes into the sparse tensor $\underline{\mathbf{\Phi}}$, we impose the constraint stated in equation (18) by applying the following normalization:

$$\underline{\mathbf{\Phi}}(:, v, f) = S_0(v) \frac{\underline{\mathbf{\Phi}}(:, v, f)}{\sum_a \underline{\mathbf{\Phi}}(a, v, f)} \qquad \forall (v, f). \tag{23}$$

2.3.1 Comparison between the multidimensional encoding framework with the standard fascicles representation

It is standard to represent fascicles as an ordered series of x, y, z coordinates. Each coordinate represents one fascicle node and each fascicle is composed of multiple nodes. Nodes are spaced anything between 0.1 to 1 mm. Our multidimensional encoding framework capture fascicles in a manner that is similar but different from the standard method. Below, we discuss the properties of the multidimensional encoding framework and advantages and potential disadvantages as compared to the standard representation.

- **Storage: Supplementary Fig. 2n** compares the computer memory required by MatLab the fascicles using our framework and the standard method. We plot: (i) the sparse core tensor $\underline{\mathbf{\Phi}}$ and (ii) the vectors of 3D points (fiber group). It is noted that, the sparse core tensor requires slightly more memory than the standard format. This is the case because whereas each fascicle node is represented in the standard format by three double precision floating-point numbers, the array encoding in MatLab uses four double precision floating point numbers. This is due to the sparse-array MatLab implementation (Tensor Toolbox[11,12]). However, as we discuss below, our tensor encoding allows for very efficient computations.

- **Efficient signal prediction model** Our first application in the manuscript shows that by using the encoding framework allows discretizing the orientations of diffusion-prediction kernels by using dictionary of pre-computed diffusion tensors. This discretization allows saving memory when predicting the signals as compared to the original LiFE model based on matrices (LiFE_M)[10].

- **Efficient computation** The advantage of our encoding method is that to represent in a compact form the integration of the dMRI signal, the streamlines and the LiFE model. By virtue of this integration, the sparse tensor representation makes forward model calculations (based either on the fascicles, data or model) more straightforward by allowing access to streamlined tensor operations, such as direct indexing operations. This is illustrated in the third and fourth application in the manuscript.

- **Sub-voxel information** The encoding framework loses some information on the position of the fascicles within a voxel. Indeed, when mapping fascicles-nodes to locations in the array $\underline{\mathbf{\Phi}}$ nodes are assumed to be centered in each voxel. This approach simplifies most mapping calculations. In practice, the effect of this assumption depends on multiple properties of the data, such as its resolution and SNR. Future advances in measurement to improve data SNR or resolution are likely to mitigate the effects of this assumption.

- **Fascicle nodes order** The sparse tensor representation loses the information about the order of each node in a fascicle. However, when needed this information can be conveniently mapped using separated vectors storing nodes order. In addition, in theory the $\underline{\mathbf{\Phi}}$ array could in principle be extended by adding one dimension to map the node order.

2.4 Fitting the LiFE_T model

Once the LiFE_T has been built, the final step to validate a connectome requires finding the non-negative weights that least-square fit the measured diffusion data. This is a convex optimization problem that can be solved using a variety of Non-Negative Least Squares (NNLS) optimization algorithms. We used a NNLS algorithm based on first-order methods specially designed for large scale problems[13]. Hereafter, we show how to exploit the decomposed LiFE_T model in the optimization.

The gradient of the original objective function for the LiFE model can be written as follows:

$$\nabla_{\mathbf{w}}\left(\frac{1}{2}\|\mathbf{y}-\mathbf{M}\mathbf{w}\|^2\right) = \mathbf{M}^T\mathbf{M}\mathbf{w} - 2\mathbf{M}^T\mathbf{y}, \tag{24}$$

where $\mathbf{M} \in \mathbb{R}^{N_\theta N_v \times N_f}$ is the original LiFE model, $\mathbf{w} \in \mathbb{R}^{N_f}$ the fascicle weights and $\mathbf{y} \in \mathbb{R}^{N_\theta N_v}$ the demeaned diffusion signal. Because the decomposed version does not explicitly store $\mathbf{M}$, below we describe how to perform two basic operations ($\mathbf{y} = \mathbf{M}\mathbf{w}$ and $\mathbf{w} = \mathbf{M}^T\mathbf{y}$) using the sparse decomposition.

2.4.1 *Computing* $\mathbf{y} = \mathbf{M}\mathbf{w}$

The product $\mathbf{M}\mathbf{w}$ can be computed in the following way using a tensor by vector product:

$$\mathbf{Y} = \underline{\mathbf{M}} \times_3 \mathbf{w}^T, \tag{25}$$

where the result is a matrix $\mathbf{Y} \in \mathbb{R}^{N_\theta \times N_v}$, a matrix version of the vector $\mathbf{y}$. Using the LiFE$_\mathrm{T}$ model the product is written as follows:

$$\mathbf{Y} = \underline{\mathbf{\Phi}} \times_1 \mathbf{D} \times_3 \mathbf{w}^T. \tag{26}$$

In **Algorithm 1**, we present the steps for computing $\mathbf{y} = \mathbf{M}\mathbf{w}$ in an efficient way.

Algorithm 1 : y = M_times_w($\underline{\mathbf{\Phi}}$,D,w)

Require: Decomposition components ($\underline{\mathbf{\Phi}}$, $\mathbf{D}$ and vector $\mathbf{w} \in \mathbb{R}^{N_f}$.
Ensure: $\mathbf{y} = \mathbf{M}\mathbf{w}$
$\mathbf{Y} = \underline{\mathbf{\Phi}} \times_3 \mathbf{w}^T$; `the result is a large but very sparse matrix` $(N_a \times N_v)$
$\mathbf{Y} = \mathbf{D}\mathbf{Y}$; `the result is a relatively small matrix` $(N_\theta \times N_v)$
$\mathbf{y} = vec(\mathbf{Y})$
return $\mathbf{y}$;

2.4.2 *Computing* $\mathbf{w} = \mathbf{M}^T\mathbf{y}$

The product $\mathbf{w} = \mathbf{M}^T\mathbf{y}$ can be computed using LiFE$_\mathrm{T}$ in the following way:

$$\mathbf{w} = \mathbf{M}^T\mathbf{y} = \mathbf{M}_{(3)}\mathbf{y} = \mathbf{\Phi}_{(3)}(\mathbf{I} \otimes \mathbf{D}^T)\mathbf{y}, \tag{27}$$

where $\otimes$ is the Kronecker product and $\mathbf{I}$ is the $(N_v \times N_v)$ identity matrix. Equation (27) can be written also as follows[4]:

$$\mathbf{w} = \mathbf{\Phi}_{(3)} vec(\mathbf{D}^T\mathbf{Y}), \tag{28}$$

where $vec()$ stands for the vectorization operation, i.e. to convert a matrix to a vector by stacking its columns in a long vector.

Because matrix $\mathbf{\Phi}_{(3)}$ is very sparse, we avoid computing the large and dense matrix $\mathbf{D}^T\mathbf{Y}$ and compute instead only its blocks that are being multiplied by the non-zero entries in $\mathbf{\Phi}_{(3)}$. This allows maintaining efficient memory usage and limits the necessary number of CPU cycles. In **Algorithm 2**, we present the steps for computing $\mathbf{w} = \mathbf{M}^T\mathbf{y}$ in an efficient way.

Algorithm 2 : w = Mtransp_times_y($\underline{\mathbf{\Phi}}$,D,y)

Require: Decomposition components ($\underline{\mathbf{\Phi}}$, $\mathbf{D}$) and vector $\mathbf{y} \in \mathbb{R}^{N_\theta N_v}$.
Ensure: $\mathbf{w} = \mathbf{M}^T\mathbf{y}$
$\mathbf{Y} \in \mathbb{R}^{N_\theta \times N_v} \leftarrow \mathbf{y} \in \mathbb{R}^{N_\theta N_v}$; `reshape vector` $\mathbf{y}$ `into a matrix` $\mathbf{Y}$
$[\mathbf{a}, \mathbf{v}, \mathbf{f}, \mathbf{c}]$ = get_nonzero_entries($\underline{\mathbf{\Phi}}$); $a(n)$, $v(n)$, $f(n)$, $c(n)$ indicate the atom, the voxel, the fascicle and coefficient associated to node n, respectively, with $n = 1, 2, \ldots, N_n$;
$\mathbf{w} = \mathbf{0} \in \mathbb{R}^{N_f}$; `Initialize weights with zeros`
for $n = 1$ **to** N_n **do**
  $w(f(n)) = w(f(n)) + \mathbf{D}^T(:, a(n))\mathbf{Y}(:, v(n))c(n)$;
end for
return $\mathbf{w}$;

2.5 Analysis of the LiFE$_T$ model accuracy

The LiFE$_T$ model provides an approximation of the original LiFE model. In this section we derive a theoretical upper bound for the model approximation error $\mathbf{e_M}$ defined in **Methods**, equation (12) as a function of the discretization parameter L, such as $\lim_{L\to\infty} \mathbf{e_M} = 0$

Let us start by focussing in the approximation error of LiFE$_T$ model compared to the original LiFE model for a particular voxel v, fascicle f and gradient direction $\boldsymbol{\theta}$. In this case, the error in modeling the diffusion signal is given by:

$$\Delta_\mathbf{O} = |\mathbf{O}_f(\boldsymbol{\theta}) - \mathbf{D}(\boldsymbol{\theta}, a)|, \tag{29}$$

where $\mathbf{O}_f(\boldsymbol{\theta})$ is the diffusion signal as defined in **Methods**, equation (6) (we avoided making reference to the voxel v for clearity) and $\mathbf{D}(\boldsymbol{\theta}, a)$, defined in equation (17), is the diffusion signal of atom a at gradient direction $\boldsymbol{\theta} = [\theta_x, \theta_y, \theta_z]^T$. By defining $\mathbf{v} = [v_x, v_y, v_z]^T$ and $\mathbf{v}_a = \mathbf{v} + \Delta_\mathbf{v} = [v_x + \Delta_{v_x}, v_y + \Delta_{v_y}, v_z + \Delta_{v_z}]^T$ as the vectors pointing out at the directions of the fascicle f and its closest dictionary atom a, respectively (see **Supplementary Fig. 2f**), and using the "stick" model diffusion tensor, i.e. $s_a = 1$ and $s_{r_1} = s_{r_1} = 0$ in equation (14), the diffusion tensors of the associated fascicle f and its approximation are:

$$\mathbf{Q}_f = \mathbf{v}\mathbf{v}^T \quad \text{and} \quad \mathbf{Q}_a = (\mathbf{v} + \Delta_\mathbf{v})(\mathbf{v} + \Delta_\mathbf{v})^T. \tag{30}$$

Now, using equations (6), (17) and (30) into equation (29), we arrive at:

$$\Delta_\mathbf{O} = |\Delta_f - \frac{1}{N_\theta} \sum_\theta \Delta_f|, \tag{31}$$

where $\Delta_f = |f(\mathbf{v}_1 + \Delta_\mathbf{v}) - f(\mathbf{v})|$ with $f(\mathbf{v}) = e^{-b(\boldsymbol{\theta}^T\mathbf{v})^2}$.

For a sufficiently small error vector $\Delta_\mathbf{v} = [\Delta_{v_x}, \Delta_{v_y}, \Delta_{v_z}]^T$, we can approximate Δ_f as follows:

$$\Delta_f \approx \left|\frac{\partial f}{\partial v_x}\right| \Delta_{v_x} + \left|\frac{\partial f}{\partial v_y}\right| \Delta_{v_y} + \left|\frac{\partial f}{\partial v_z}\right| \Delta_{v_z}, \tag{32}$$

$$\approx 2b|\boldsymbol{\theta}^T\mathbf{v}|e^{-b(\boldsymbol{\theta}^T\mathbf{v})^2}(|\theta_x\Delta_{\mathbf{v}_x}| + |\theta_y\Delta_{\mathbf{v}_y}| + |\theta_z\Delta_{\mathbf{v}_z}|). \tag{33}$$

By using the fact that $|\boldsymbol{\theta}^T\mathbf{v}| \leq 1$, $e^{-b(\boldsymbol{\theta}^T\mathbf{v})^2} \leq 1$, $\Delta_{v_x}, \Delta_{v_y}, \Delta_{\mathbf{v}_z} \leq \|\Delta_\mathbf{v}\| \leq \frac{\pi}{\sqrt{2}L}$, and $\|\boldsymbol{\theta}\|_1 \leq \sqrt{3}\|\boldsymbol{\theta}\|$, we obtain:

$$\Delta_f \leq \frac{b\pi\sqrt{6}}{L}. \tag{34}$$

Finally, by using the equation (34) into equation (31), we obtain an upper bound for the error modeling the diffusion signal of one fascicle f at one gradient direction in a voxel:

$$\Delta_\mathbf{O} \leq \frac{2b\pi\sqrt{6}}{L}. \tag{35}$$

In order to establish a theoretical upper bound of the model error ($\mathbf{e_M}$, **Methods**, equation 12) as a function of the discretization parameter L, we need to find an upper bound of $\|\mathbf{M} - \hat{\mathbf{M}}\|$ and note $\|\mathbf{M}\|$ is independent of the discretization parameter L. The upper bound of $\|\mathbf{M} - \hat{\mathbf{M}}\|^2$ can be obtained by assuming that all fascicles are composed of a fixed number of nodes N_n, and adding up over all nodes n, fascicles f and directions $\boldsymbol{\theta}$, i.e.

$$\|\mathbf{M} - \hat{\mathbf{M}}\|_F^2 \leq N_f N_n N_{\boldsymbol{\theta}} \left(\frac{2b\pi\sqrt{6}}{L}\right)^2. \tag{36}$$

Finally, using equation (36) in equation (12) we obtain the following upper bound of the model error:

$$\mathbf{e_M} \leq \frac{\kappa}{L}, \tag{37}$$

where $\kappa = \frac{\sqrt{6N_f N_n N_{\boldsymbol{\theta}}}2b\pi}{\|\mathbf{M}\|_F}$ is a constant (independent of discretization parameter L).

Equation (37) clearly states that the achievable relative error is inversely proportional to the discretization parameter L, which allows us to make the model as accurate as we want by just increasing the dictionary resolution, i.e. increasing L.

Finally, we note that this discretization of the diffusion-orientation prediction on the sphere (**Supplementary Fig. 2f**) is non-uniform, which in turns leads to a denser discretization around the poles of the sphere. This is the result of using a uniform sampling along the dimensions of azimuth and elevation. This choice improves efficiency in mapping fascicles-nodes to dictionary ($\mathbf{D}$) and sparse array ($\underline{\mathbf{\Phi}}$) entries.This is because we can use an analytic solution to compute the spherical coordinates and map the orientation of each fiber at each node onto the sphere, and the corresponding columns in $\mathbf{D}$ and entries in $\underline{\mathbf{\Phi}}$.

Yet, because of the non-uniform sampling on the sphere, it is in principle possible that the LiFE_T model could be biased toward supporting more fascicles closer aligned to the poles of the spheres in $\mathbf{D}$. THis is because at orientation closer to the poles the dictionary resolution is highest. We performed an explicit test to show whether the discretization biases the model fit. We compared our original results, both r.m.s error and fascicle weights ($\mathbf{w}$), with new ones obtained by rotating the dMRI image and fascicles 90 degrees (on the axis x) without rotating dictionary. This manipulation changes the position of the higher-density sphere sampling poles in relation to the fascicles and dMRI data. If the density affects the model fit we expect either r.m.s. or w to differ. The test was performed on 8 subjects (HCP3T and STN) using probabilistic tractography (L_{max} = 10) and using dictionaries with the fixed density (that is $L = 360$, corresponding to about $N_a = 129,241$ dictionary entries). The results of this test are presented in **Supplementary Fig. 2o**. The results demonstrate a very small effect of the rotation, with measured less than 0.5% change in r.m.s. error and less than 0.05% change in the assigned weights.

2.6 Analysis of the LiFE_T model compression factor

Here, we analytically derive the storage requirements of matrix $\mathbf{M}$ in LiFE (**Supplementary Fig. 2c**;[10]) and its approximation $\hat{\mathbf{M}}$ through LiFE_T, decomposed model (**Supplementary Fig. 2d**). To do so, as in the previous section, we simplify the analysis by assuming that all fascicles have the same number of nodes N_n and that there are no more than one node per fascicle, per voxel. Under these ideal assumptions the amount of memory necessary to store each fascicle f in a sparse matrix $\mathbf{M}$ is $3N_{\boldsymbol{\theta}}N_n$, since using a sparse matrix structure, three numbers are required for each node, i.e. the row-column indices plus the entry value. Thus the storage cost of $\mathbf{M}$ is:

$$C(\mathbf{M}) = \mathcal{O}(3N_nN_{\boldsymbol{\theta}}N_f). \tag{38}$$

Conversely, storing fascicles in the LiFE_T model requires $4N_n$ values per fascicle plus the dictionary matrix (i.e. the set of the non-zero entries and their locations within the tensor $\underline{\mathbf{\Phi}}$ together with matrix $\mathbf{D}$). Thus the amount of memory required in LiFE_T model is:

$$C(\underline{\hat{\mathbf{M}}}) = \mathcal{O}(4N_nN_f + N_{\boldsymbol{\theta}}N_a), \tag{39}$$

where $N_{\boldsymbol{\theta}}N_a$ is the storage associated with the dictionary matrix $\mathbf{D} \in \mathbb{R}^{N_{\boldsymbol{\theta}} \times N_a}$. The Compression Factor can be straightforwardly computed as follows:

$$CF = \left(\frac{4}{3N_{\boldsymbol{\theta}}} - \frac{N_a}{3N_nN_f}\right)^{-1}. \tag{40}$$

Given that, usually $3N_nN_f \gg N_a$, the compression factor can be estimated[b] as follows:

$$CF \approx \frac{3N_{\boldsymbol{\theta}}}{4}. \tag{41}$$

Equation (41) states that the compression factor is proportional to the number of directions $N_{\boldsymbol{\theta}}$, which represents a substantial reduction in memory requirements for modern datasets.

[b] We note that our experimental results in **Fig. 2d** showed a compression factor slightly lower than the theoretical estimation because the sparse matrix format implemented in Matlab[14] is relatively more efficient than the sparse array format used in the Matlab Tensor Toolbox[12]

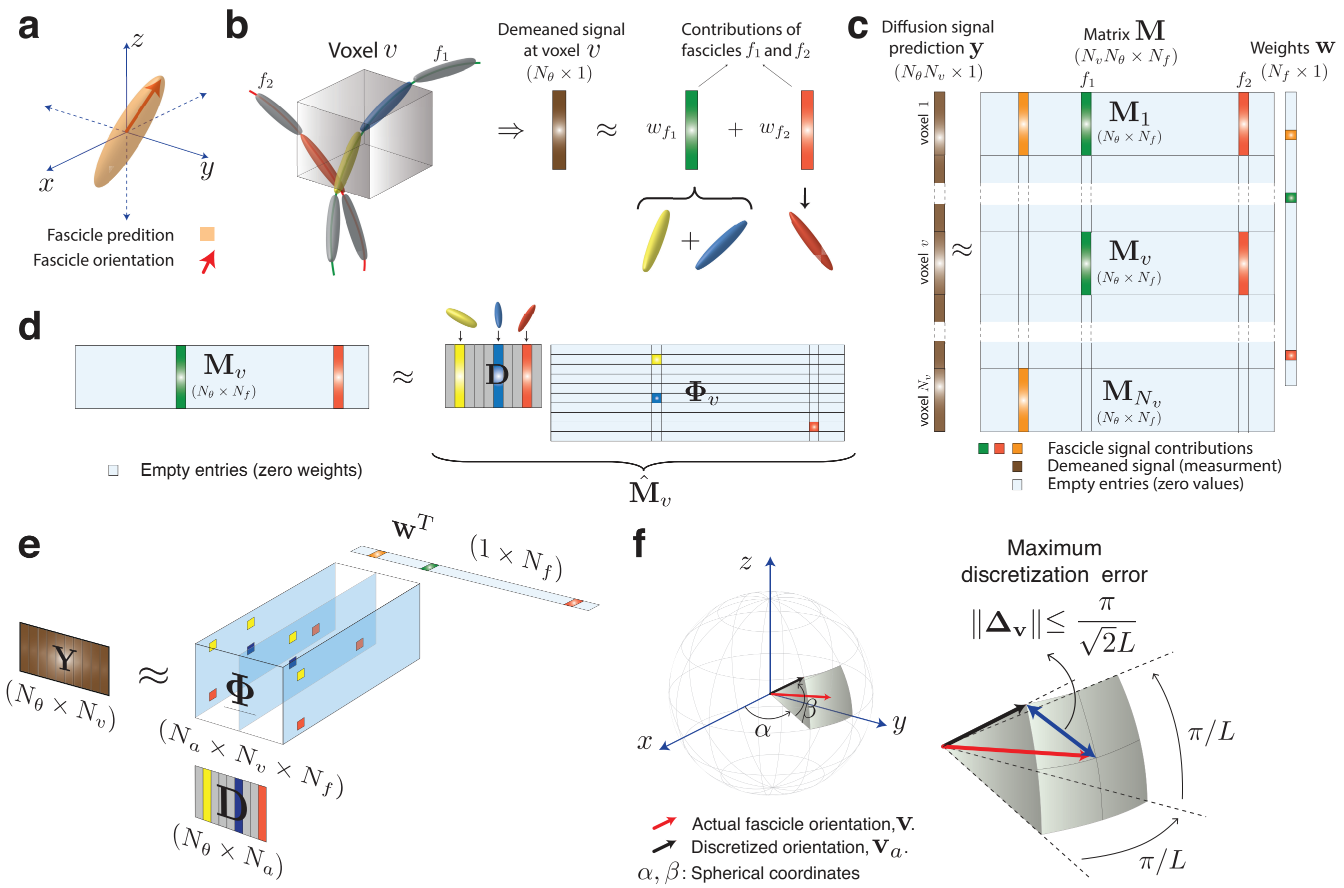

Supplementary Fig. 2. **(a)** A diffusion tensor model whose principal axes are given by the spatial orientation of a fascicle in a voxel v. **(b)** Linear model at voxel level: diffusion signal prediction for a voxel intersected by two fascicles, f_1 and f_2, is shown. Fascicle f_1 crosses the voxel from one side to the other and it bends within it, which is modeled as composed by two nodes (yellow and blue). Fascicle f_2 occupies a small portion of the voxel contributing with one diffusion tensor (red). The total prediction signal is the linear combination of the signals associated to existing fascicles within the voxel. **(c)** Linear model for all voxels: LiFE model matrix $\mathbf{M}$[10] is obtained by stacking in its columns, the diffusion signal predictions of fascicles. The predicted diffusion signal in all voxels is obtained as $\mathbf{y} \approx \mathbf{M}\mathbf{w}$, where $\mathbf{w}$ is a sparse vector of weights obtained by solving a convex optimization problem with non-negativity constraints. **(d)** Sparse factorization of the diffusion signal in a voxel: Nonzero columns in block matrix $\mathbf{M}_v$ correspond to fascicles passing through v (two in our example). Those columns are approximated by combining the diffusion prediction of the dictionary atoms (columns of matrix $\mathbf{D}$) with directions closest to the orientation of the fascicles nodes (yellow, blue and red). Non-zero entries in matrix $\mathbf{\Phi}_v$ indicate the atoms corresponding to the nodes in the fascicles f_1 and f_2 in the example. **(e)** The LiFE$_\text{T}$ model: the diffusion signal matrix $\mathbf{Y} \in \mathbb{R}^{N_\theta \times N_v}$ (directions $\times$ voxels) is written as a Sparse Tucker Decomposition by using the core Sparse tensor $\underline{\mathbf{\Phi}} \in \mathbb{R}^{N_a \times N_v \times N_f}$, multiplied in mode-1 by the dictionary matrix $\mathbf{D} \in \mathbb{R}^{N_\theta \times N_a}$ and, in mode-3, by the vector of weights $\mathbf{w} \in \mathbb{R}^{N_f}$. **(f)** Error introduced by the discretization: Left, a regular grid in the sphere is obtained by discretizing the space in spherical coordinates (α, β). Right, the maximum distance between the fascicle orientation vector $\mathbf{v}$ and its approximation $\mathbf{v}_a$ is inversely proportional to the parameter L, i.e. $\|\Delta_\mathbf{v}\| \leq \frac{\pi}{\sqrt{2}L}$.

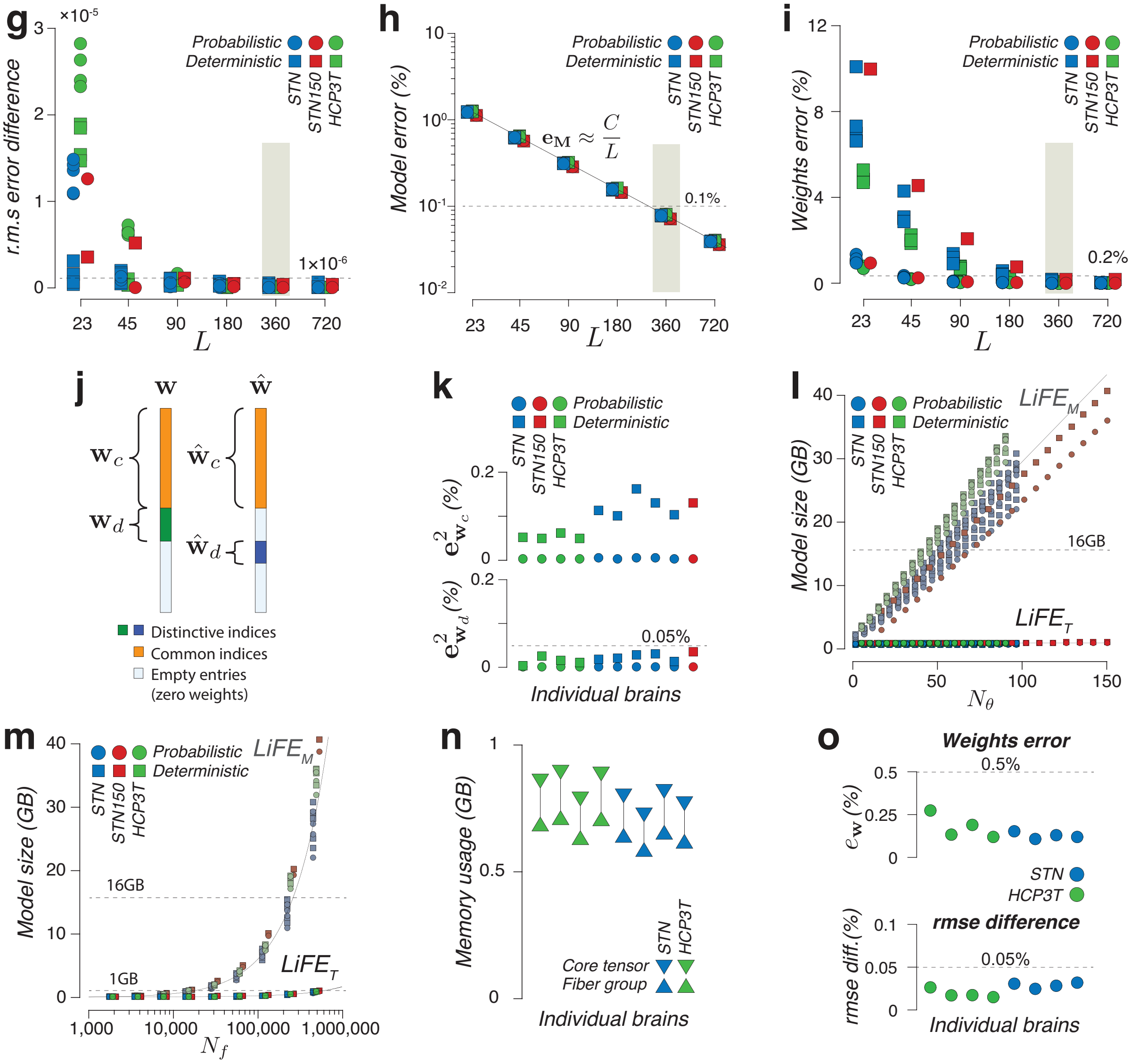

Supplementary Fig. 2. **(g)** Comparison of the global r.m.s error obtained by LiFE and LiFE$_T$ models. It is clear that for $L \geq 180$ LiFE$_T$ approximates very well the original LiFE model (difference $< 1 \times 10^{-6}$). **(h)** Weights error $e_\mathbf{w}$ versus parameter L. This plot shows that for $L \geq 360$ LiFE$_T$ provides a very good approximation of the LiFE model ($e_\mathbf{w} < 0.2\%$). **(i)** The model error $e_\mathbf{M}$ in approximating the matrix $\mathbf{M}$ with LiFE$_T$ is inversely proportional to the parameter L as predicted by our theoretical bound (see equation 37). The function $\mathbf{e_M} \approx \frac{C}{L}$ was fitted to the data, which resulted in an estimated value $C = 28.22$, and a fitting error equal to 3.03% (relative root squared error). It is noted that, for $L \geq 360$, we obtained a model error smaller than 0.1%. **(j)** By defining common (orange) and different (green and blue) subsets of indices within vectors $\mathbf{w}$ and $\hat{\mathbf{w}}$, the squared weights error is decomposed as the sum of the squared errors associated to the common and different fascicles (see equation 21). **(k)** The squared error for common fascicles $e^2_{\mathbf{w}_c}$ (top) and different fascicles $e^2_{\mathbf{w}_d}$ (bottom) computed for STN, STN150 and HCP3T datasets are shown. While the total squared $e^2_\mathbf{w}$ is kept below 0.2%, it is highlighted that it is larger in the deterministic case compared against to the probabilistic case. However, the error associated to the different fascicles is very small compared to the common fascicles, which means that essentially both models use almost the same subset of fascicles. **(l)** and **(m)** Model size (GB) scales linearly with the number of directions N_θ and the number of fascicles N_f, however it increases much faster in the LiFE model compared to the LiFE$_T$ model. **(n)** Comparison of memory usage in Matlab to store a connectome as a fiber group (points in the 3D space) and as a sparse core tensor $\underline{\boldsymbol{\Phi}}$ computed on 8 subjects (HCP3T and STN) using probabilistic tractography ($L_{max} = 10$). It is noted that, even that the core tensor requires moderately more memory, our decomposed tensor model provides the information on the relation between fascicles and diffusion signal. **(o)** To test the existence of a bias due to the non uniform distribution of dictionary elements, we computed the relative error on the weights $e_\mathbf{w}$ and difference in rmse values (defined as $\|\mathbf{e}_{rms} - \hat{\mathbf{e}}_{rms}\| / \|\mathbf{e}_{rms}\|$) by comparing the results with the one obtained by rotating 90 degrees the diffusion data and the fascicles. These results show that the difference is very small (less than 0.5 % and 0.05 % for the weights and rmse, respectively).

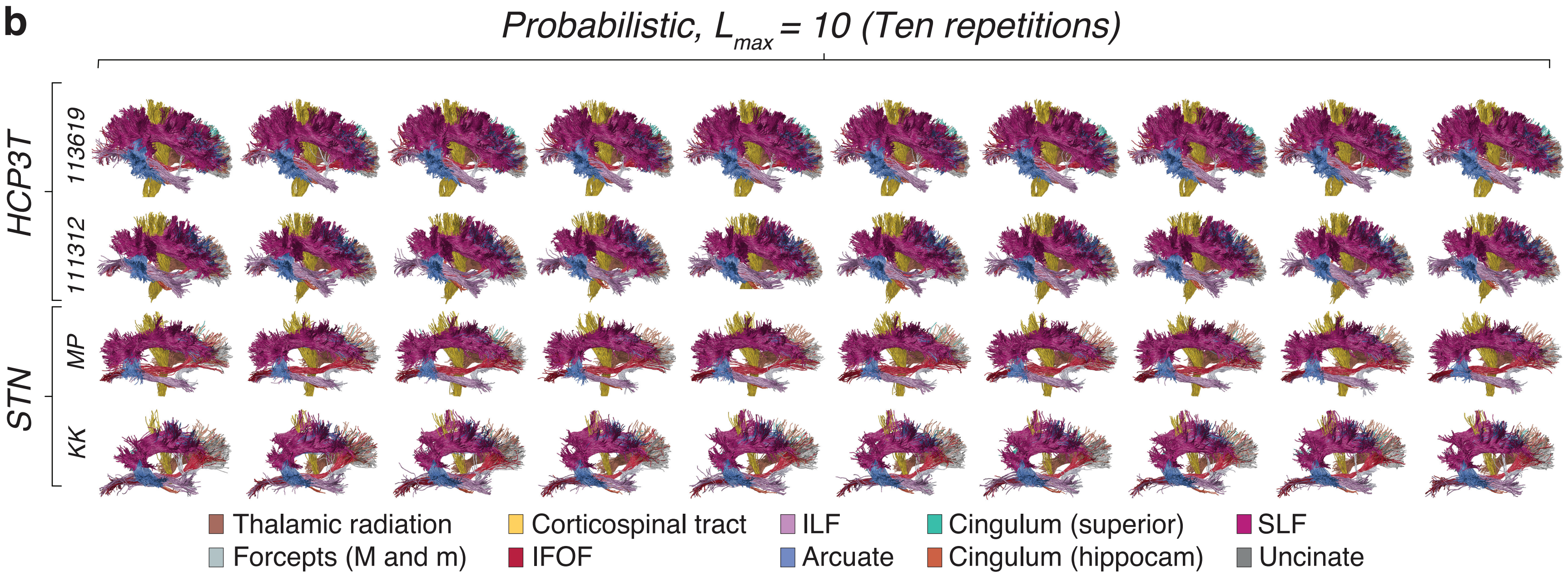

Supplementary Fig. 3. (**b**) Major human tracts from LiFE optimized connectomes (fascicles with nonzero weights) using Probabilistic tractography ($L_{max} = 10$) for two subjects in the STN dataset and two subjects in the HCP3T dataset. Results obtained by repeating the tractography and optimization ten times are shown in different columns. It is highlighted that connectomes are anatomically discriminable across subjects and datasets (rows) but preserving the anatomy among repetitions of the LiFE evaluation (columns).

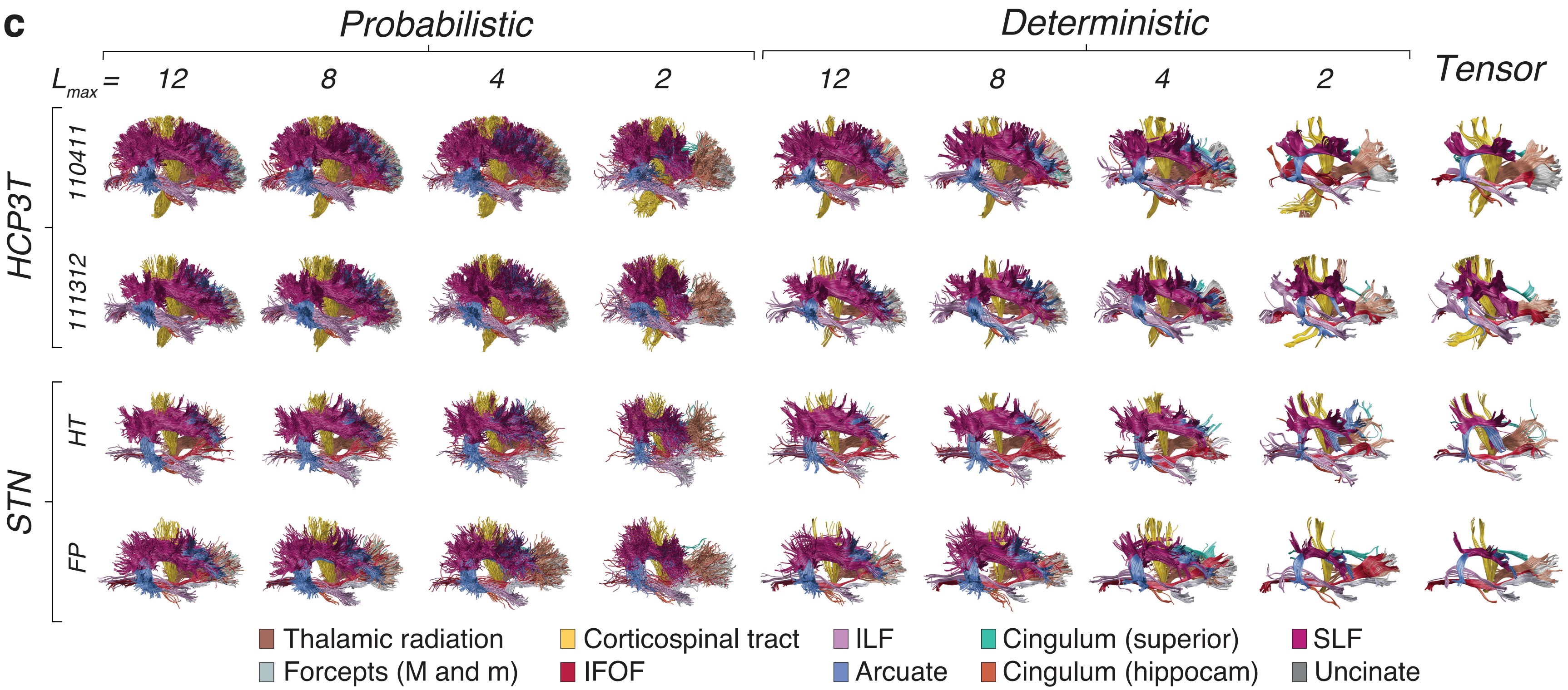

Supplementary Fig. 3. **(c)** Major human tracts from LiFE optimized connectomes (fascicles with nonzero weights) using probabilistic ($L_{max} = 2, 4, 6, 8, 10, 12$), deterministic ($L_{max} = 2, 4, 6, 8, 10, 12$) and tensor tracking methods for two subjects in the STN dataset and two subjects in the HCP3T dataset. It is highlighted that connectomes are anatomically discriminable across subjects and datasets (rows) and across tracking methods.

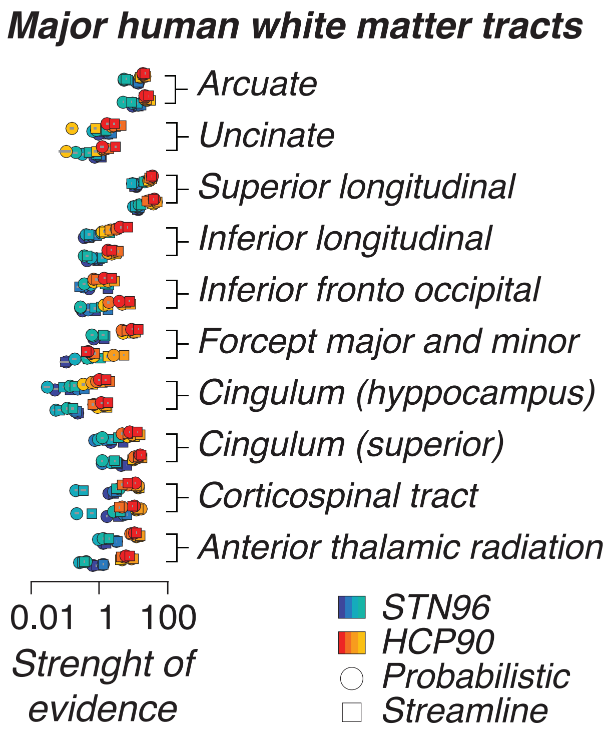

Supplementary Fig. 4. The strength of evidence S is a measure of distance between the distributions of r.m.s errors in voxels for the model with virtual lesion and without virtual lesion (see[10] for details). Similarly to the Earth Mover Distance measure (**Fig. 4d**), the strength of evidence is positive for the major tracts, which replicates the statistical evidence of major tracts reported in[10].

References

1. Kolda, T. & Bader, B. Tensor decompositions and applications. *SIAM Review* **51**, 455–500 (2009).
2. Cichocki, A. *et al.* Tensor decompositions for signal processing applications: from two-way to multiway component analysis. *IEEE Signal Processing Magazine* **32**, 145–163 (2015).
3. Tucker, L. R. Some mathematical notes on three-mode factor analysis. *Psychometrika* **31**, 279–311 (1966).
4. Caiafa, C. F. & Cichocki, A. Computing Sparse representations of multidimensional signals using Kronecker bases. *Neural Computation* 186–220 (2012).
5. Caiafa, C. F. & Cichocki, A. Multidimensional compressed sensing and their applications. *Wiley Interdisciplinary Reviews: Data Mining and Knowledge Discovery* **3**, 355–380 (2013).
6. Assaf, Y. & Basser, P. J. Composite hindered and restricted model of diffusion (CHARMED) MR imaging of the human brain. *NeuroImage* **27**, 48–58 (2005).
7. Frank, L. R. Characterization of anisotropy in high angular resolution diffusion-weighted MRI. *Magnetic Resonance in Medicine* **47**, 1083–1099 (2002).
8. Behrens, T. E. J. *et al.* Characterization and propagation of uncertainty in diffusion-weighted MR imaging. *Magnetic Resonance in Medicine* **50**, 1077–1088 (2003).
9. Basser, P., Mattiello, J. & Lebihan, D. Estimation of the effective self-diffusion tensor from the NMR spin echo. *Journal of Magnetic Resonance, Series B* **103**, 247–254 (1994).
10. Pestilli, F., Yeatman, J. D., Rokem, A., Kay, K. N. & Wandell, B. A. Evaluation and statistical inference for human connectomes. *Nature Methods* **11**, 1058–1063 (2014).
11. Bader, B. W. & Kolda, T. G. Efficient MATLAB Computations with Sparse and Factored Tensors. *SIAM J. SCI. COMPUT.* **30**, 205–231 (2008).

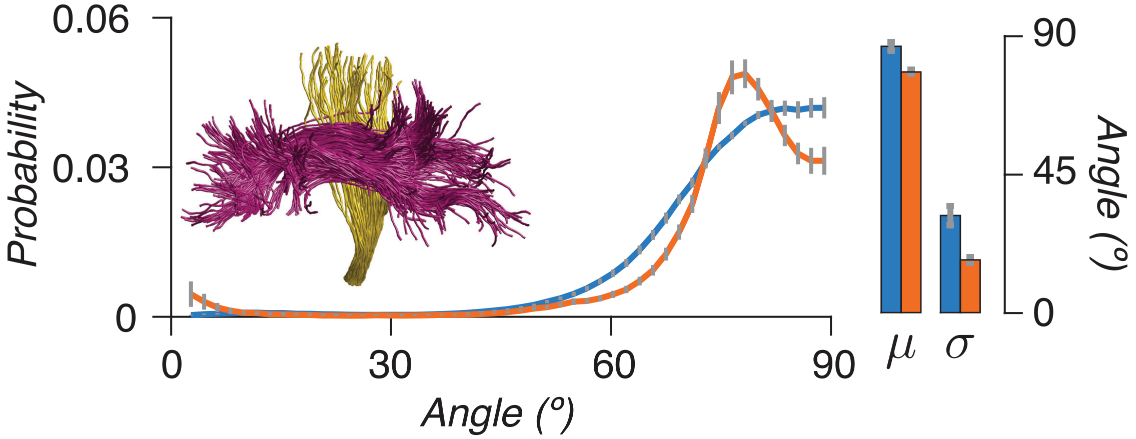

Supplementary Fig. 5. The histograms of angles between fascicles in the Corticospinal tract and SLF tracts are shown for probabilistic (blue) and deterministic (orange) tracking methods with $L_{max} = 10$. These distributions show a crossing close to perpendicular similar to the findings for the case of Arcuate and Corticospinal tract in **Fig. 5e**.

12. Bader, B. W., Kolda, T. G. & others. MATLAB Tensor Toolbox Version 2.5. Tech. Rep., Sandia National Laboratories (2012).

13. Kim, D., Sra, S. & Dhillon, I. S. A non-monotonic method for large-scale non-negative least squares. *Optimization Methods and Software* **28**, 1012–1039 (2013).

14. Gilbert, J. R., Moler, C. & Schreiber, R. Sparse matrices in matlab: design and implementation. *SIAM Journal on Matrix Analysis and Applications* **13**, 333–356 (1992).
